# Extended characterisation of five archival tick-borne viruses provides insights for virus discovery in Australian ticks

**DOI:** 10.1186/s13071-022-05176-z

**Published:** 2022-02-18

**Authors:** Caitlin A. O’Brien, Bixing Huang, David Warrilow, Jessamine E. Hazlewood, Helle Bielefeldt-Ohmann, Sonja Hall-Mendelin, Cassandra L. Pegg, Jessica J. Harrison, Devina Paramitha, Natalee D. Newton, Benjamin L. Schulz, Andreas Suhrbier, Jody Hobson-Peters, Roy A. Hall

**Affiliations:** 1 School of Chemistry and Molecular Biosciences, The University of Queensland, St. Lucia, QLD 4072 Australia; 2 Australian Infectious Disease Research Centre, GVN Center of Excellence, The University of Queensland and QIMR Berghofer Medical Research Institute, St Lucia, QLD 4067 Australia; 3 Public Health Virology, Forensic and Scientific Services, Department of Health, P.O. Box 594, Archerfield, QLD Australia; 4 Inflammation Biology Group, QIMR Berghofer Medical Research Institute, Brisbane, QLD 4006 Australia; 5 School of Veterinary Science, The University of Queensland, Gatton, QLD Australia

**Keywords:** *Ixodes holocyclus*, *Ixodes uriae*, Tick-borne flavivirus, Tick-borne orbivirus, *Nairoviridae*, Tick-borne phlebovirus, Virus discovery

## Abstract

**Background:**

A subset of Australians who have been bitten by ticks experience a complex of chronic and debilitating symptoms which cannot be attributed to the known pathogenic species of bacteria present in Australia. As a result, there has been a renewed effort to identify and characterise viruses in Australian terrestrial ticks. Recent transcriptome sequencing of *Ixodes* and *Amblyomma* ticks has revealed the presence of multiple virus sequences. However, without virus isolates our ability to understand the host range and pathogenesis of newly identified viruses is limited. We have established a successful method for high-throughput virus discovery and isolation in mosquitoes using antibodies to double-stranded RNA. In this study we sought to characterise five archival tick-borne viruses to adapt our virus discovery protocol for Australian ticks.

**Methods:**

We performed virus characterisation using a combination of bioinformatic sequence analysis and in vitro techniques including replication kinetics, antigenic profiling, virus purification and mass spectrometry.

**Results:**

Our sequence analysis of Nugget virus, Catch-me-Cave virus and Finch Creek virus revealed marked genetic stability in isolates collected from the same location approximately 30 years apart. We demonstrate that the *Ixodes scapularis*-derived ISE6 cell line supports replication of Australian members of the *Flaviviridae*, *Nairoviridae*, *Phenuiviridae* and *Reoviridae* families, including Saumarez Reef virus (SREV), a flavivirus isolated from the soft tick *Ornithodoros capensis*. While antibodies against double-stranded RNA could be used to detect replication of a tick-borne reovirus and mosquito-borne flavivirus, the tick-borne flaviviruses Gadgets Gully virus and SREV could not be detected using this method. Finally, four novel virus-like sequences were identified in transcriptome sequencing of the Australian native tick *Ixodes holocyclus.*

**Conclusions:**

Genetic and antigenic characterisations of archival viruses in this study confirm that three viruses described in 2002 represent contemporary isolates of virus species first identified 30 years prior. Our findings with antibodies to double-stranded RNA highlight an unusual characteristic shared by two Australian tick-borne flaviviruses. Finally, comparative growth kinetics analyses of Australian tick-borne members of the *Flaviviridae*, *Nairoviridae*, *Phenuiviridae* and *Reoviridae* families in ISE6 and BSR cells will provide a useful resource for isolation of Australian tick-borne viruses using existing cell lines.

**Graphical Abstract:**

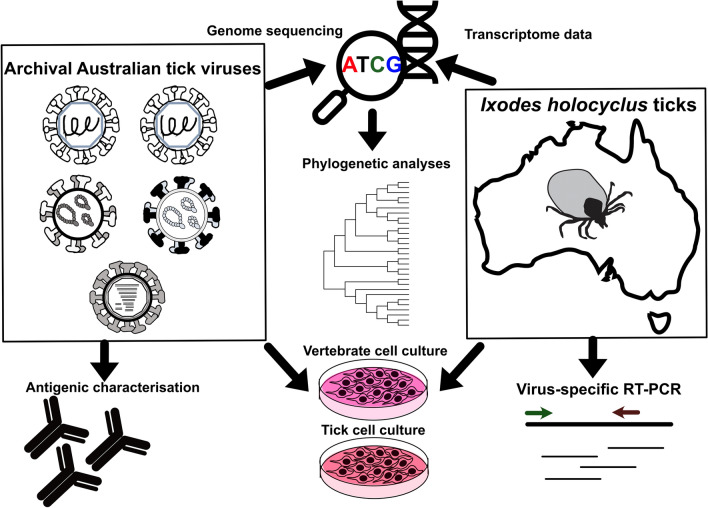

**Supplementary Information:**

The online version contains supplementary material available at 10.1186/s13071-022-05176-z.

## Background

Although Australia is home to a range of mosquito-borne viral pathogens [1], at present there are no recognised tick-borne viral diseases [2]. This is at odds with the rest of the world where tick-borne viruses pose significant threats to both human and animal health [3–7]. Despite this, a subset of Australians live with a chronic, incapacitating syndrome now termed “Debilitating Symptom Complexes Attributed to Ticks” (DSCATT), which cannot be explained by the known tick-borne bacterial pathogens found in Australia [2]. With the recognition of DSCATT, a focus of research has moved towards identifying potential emerging pathogens in Australian terrestrial ticks.

**Fig. 1:**
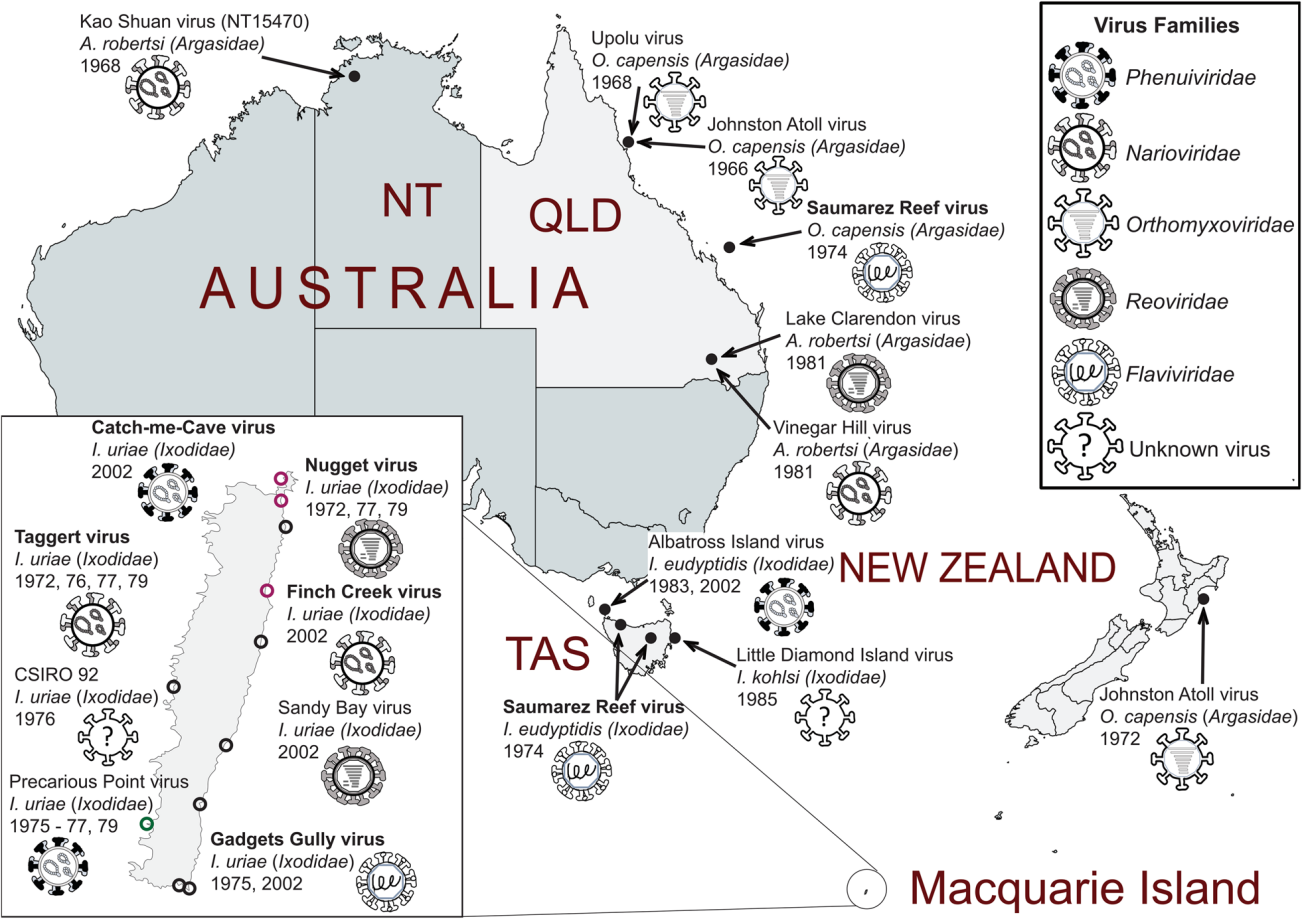
Schematic of tick viruses isolated in the Australia and New Zealand region. The tick species (family) of isolate and year of collection are indicated beneath the name of each virus. The viral family of each isolate is indicated by icons defined in the legend on the right-hand side of the figure. Bold text indicates viruses used in this study. Inset, summary of viruses isolated on Macquarie Island. Circled regions indicate sites where tick collections yielded virus isolates. Black circles, collection sites accessed between 1972 and 1979. Magenta circles, collection sites accessed in 2002. Green circle, Precarious Point where tick collections occurred both in the 1970s and 2002. Tick genus names: *A*., *Argas*; *I*., *Ixodes*; *O*., *Ornithodoros*

Australia is home to at least 70 species of ticks, many of which are unique to the continent [8]. However, migratory birds also carry ticks which are found elsewhere in the world including *Ixodes uriae*, *Argas robertsi* and *Ornithodoros capensis*. The search for tick-borne viruses in Australia was first initiated in the 1970s by the Commonwealth Science and Industrial Research Organisation (CSIRO). This work yielded isolates belonging to at least five different viral families (*Nairoviridae*, *Phenuiviridae*, *Flaviviridae*, *Reoviridae*, *Orthomyxoviridae*), primarily from seabird-associated ticks (Fig. 1). Macquarie Island is a sub-Antarctic island located halfway between the Australian state of Tasmania and Antarctica. The island is home to large rookeries of penguins and the ticks that infest them, including the hard tick *I. uriae*. Collections of *I. uriae* from Macquarie Island between 1972 and 1979 led to the isolation of Gadgets Gully virus (GGYV, *Flaviviridae*), Precarious Point virus (PPV, *Phenuiviridae*), Taggert virus (TAGV, *Nairoviridae*) and Nugget virus (NUGV, *Reoviridae*) [9, 10]. In addition, Saumarez Reef virus (SREV, *Flaviviridae*) was isolated from seabird-associated ticks collected on Saumarez Reef off the coast of Queensland (QLD; *O. capensis*) and Tasmania (*I. eudyptidis*) in 1974 (Fig. 1) [11]. Serology-based analyses showed each of these viruses shared antigenic relatedness to tick-borne viruses of the Northern Hemisphere, some of which include human pathogens such as Kemerovo virus and tick-borne encephalitis virus [9–11]. PPV, TAGV, GGYV and SREV have since been sequenced, further supporting these links with the Northern hemisphere [12–14].

A re-survey of *I. uriae* ticks from Macquarie Island in 2002 resulted in the re-isolation of GGYV [15], approximately 30 years after its initial isolation [10]. Despite the length of time between collections, sequencing of the E and NS1 genes showed minimal genetic variation between these isolates. This study also yielded three additional arbovirus isolates, Sandy Bay virus (SBaV, *Reoviridae*), Finch Creek virus (FCV, *Nairoviridae*) and Catch-me-cave virus (CMCV, *Phenuiviridae*), for which partial genome sequence was obtained (Fig. 1) [15]. A comparison of the S segment of CMCV and PPV indicated that the two viruses were closely related; however, the L and M segment of CMCV could not be obtained. At the time of this study no genomic information was available for TAGV; however, in 2016 sequencing of this virus showed it to be very similar to the available sequence of FCV, specifically, a 392 nt region of the L segment [14].

To our knowledge the only viruses to be isolated from ticks on the Australian mainland are Lake Clarendon virus, Vinegar Hill virus (VINHV) and a strain of Kao Shuan virus all of which were derived from the soft tick *A. robertsi* (Fig. 1) [16–18]. Little is known about the pathogenicity of these archival viruses, although antibodies to VINHV and GGYV have been identified in human sera with no known reports of illness in the serum donors [17, 19]. Mostly, these viruses are not deemed to pose an imminent risk to public health or agriculture, or they are isolated from regions not populated by humans. As a result, for many of these isolates little characterisation has been undertaken since the initial reports.

Recent meta-transcriptomic surveys of ticks from New South Wales (NSW), Australia, have identified a number of novel virus sequences including Shelly Headland virus, which groups with the human pathogens Colorado tick fever virus and Eyach virus [20, 21]. While next generation sequencing technologies are an invaluable asset in the field of virus discovery, isolation of viruses of interest is necessary to further our understanding of host range and pathogenesis for potential emerging viruses [22].

We have developed a novel system for broad-spectrum identification of RNA viruses based on the detection of double-stranded RNA (dsRNA) [23]. The MAVRIC (monoclonal antibodies to viral RNA intermediates in cells) system identifies cultures positive for virus replication in a sequence-independent manner. Based on the successful implementation of this system for virus discovery in Australian mosquitoes [24–29], we assessed the utility of the MAVRIC system for virus discovery in Australian tick populations. Traditionally, arbovirus isolation has been performed on vertebrate cell lines, with Vero and BHK cells commonly used. However, even these cell lines can show varying levels of permissiveness to different viruses [26, 30]. The ISE6 cell line is a well-established cell line derived from *I. scapularis*, a tick species found in eastern USA and Canada. This cell line has been shown to support replication of a number of tick-borne viruses as well as the mosquito-borne flavivirus West Nile virus New York 99 strain [31–33]. However, the permissiveness of this cell line for isolation of viruses from native Australian ticks is unclear [34].

In this study we assessed the potential for the use of the MAVRIC system for discovery and isolation of Australian tick viruses. Using a panel of Australian archival tick viruses, we sought to further characterise the replication of these viruses and the utility of MAVRIC for Australian tick virus discovery.

## Methods

### Tick cell culture

ISE6 (*I. scapularis*) cells were maintained in complete ISE6 cell medium containing L-15B300 media supplemented with 0.1% LipiMAX bovine lipoprotein solution (Selbourne Biological, Tasmania, Australia), 10% tryptose phosphate broth (Oxoid (Thermo Fisher), Basingstoke, UK) and 5% fetal bovine serum (FBS, Bovogen, Victoria, Australia). L-15B300 medium was made in house using triple-autoclaved ultrapure water, Leibovitz-15 (L-15) media (Lonza, Gibco) and trace minerals as per [35]. The details for each reagent are described in Additional file 1: Table S1. Cells were frozen in complete medium containing 15% FBS and 10% DMSO using the CoolCell LX cell freezing container (Corning). Cells were revived in a T25 non-vented flask and maintained by replacing media every 7–10 days for 1 month before upscaling to a T75 non-vented flask. Upscaled cells were maintained for a minimum of 1 month before seeding for experiments. Cells were maintained at 34 °C without CO_2_.

### Vertebrate and mosquito cell culture

Vero (*Chlorocebus* monkey kidney cells), BHK (baby hamster kidney cells, *Mesocricetus auratus*) and BSR (clone of baby hamster kidney cell line, *Mesocricetus auratus*) cells were maintained in Dulbecco’s Modified Essential Medium (DMEM) supplemented with 5% FBS. Opossum (*Didelphidae*) kidney (OK) cells were maintained in Roswell Park Memorial Institute (RPMI) medium supplemented with 5% FBS. All vertebrate cells were maintained at 37 °C in the presence of 5% CO_2_. C6/36 cells (*Aedes albopictus*, mosquito larvae-derived) were cultured in RPMI supplemented with 5% FBS and grown at 28 °C without CO_2_.

### Production of stocks of archival viruses

Virus stocks were prepared by inoculation of vertebrate or C6/36 cells as per Table 1 at approximately 80% confluency. Where titres were known, inoculation was performed at a multiplicity of infection (MOI) of 0.1. Where virus titres in the inoculum were unknown, 100 µl of inoculum was used. After an adsorption period of 1 h at room temperature with agitation, virus inoculum was removed and replaced by DMEM (or RPMI for C6/36 cells) supplemented with 2% FBS. Cells were incubated at 37 °C for 2–7 days. Supernatant was harvested and clarified by centrifugation at 3000*g* for 15 min at 4 °C. FBS content in culture supernatant was raised to 10% prior to aliquoting and storage at − 80 °C.

**Table 1:** Virus stocks used in this study

Virus (references)	Abbreviation	Strain	Tick species	Cell line (no. days)	Passage history (cell line)
Gadgets Gully [10]	GGYV	CSIRO 122	*I. uriae*	BSR (5)	X^a^ + 2 (BHK, BSR)
Saumarez Reef [11]	SREV	CSIRO 4	*O. capensis*	BHK (3)	X + 2 (BHK)
West Nile [51]	WNV_KUN_	NSW2011	Mosquito	BHK (2)	X + 1 (BHK)
West Nile [70]	WNV_KUN_	MRM61C	Mosquito	C6/36 (5)	X + 1 (Vero)
Finch Creek [15]	FCV	EB06	*I. uriae*	Vero (6)	6 (Vero)
Taggert [9]	TAGV	MI14850	*I. uriae*	Vero (7)	X + 1 (BSR)
Catch-me-cave [15]	CMCV	I-2	*I. uriae*	Vero (5)	X + 2 (Vero)
Nugget [9]	NUGV	MI14847	*I. uriae*	BSR (7)	X + 1 (BSR)

^a^X indicates unknown number of previous passages

All viruses were titrated onto BSR and Vero cells and titres were determined by TCID_50_ using the Reed-Muench method [36]. Virus replication was determined by fixed-cell ELISA using the pan-flavivirus mAb 4G2 for SREV and GGYV, MAVRIC for NUGV and anti-FCV mouse immune serum for FCV and TAGV. Replication of CMCV was determined by the presence or absence of cytopathic effect (CPE).

### Tick homogenisation and passaging

Adult *I. holocyclus* ticks collected in Lismore, which had been blood fed on rats for an unrelated experiment, were stored at − 80 °C. Pools of five ticks were thawed on ice and added to 5 ml microcentrifuge tubes with 4 ml of chilled complete ISE6 media. Homogenisation was performed with a 5-mm stainless steel bead for 2 min, followed by centrifugation at 3000 *g* for 15 min at 4 °C to pellet debris. Clarified supernatant was passed through a 0.2-µm filter. Filter-sterilised supernatant was used immediately to inoculate cells and thereafter stored at − 80 °C. Inoculum was removed from vertebrate cells after 2 h and replaced with appropriate maintenance media before cells were incubated at 37 °C with 5% CO_2_ for 5 days post-infection (dpi). Inoculum was left on ISE6 cells for 7–9 dpi at 34 °C without CO_2_.

### RNA extraction and RT-PCR

RNA extraction was performed on homogenates and cell culture supernatant using the Machery Nagel Viral RNA isolation kit as per manufacturers’ instructions. RT-PCR testing was performed using the SuperScript™ III One-Step RT-PCR System with Platinum™ Taq DNA Polymerase (Invitrogen) and primer sets listed in Table 2. Cycling conditions for each PCR is as follows; reverse transcription: 45 °C for 30 min; PCR: one cycle 94 °C for 2 min; 40 cycles 94 °C for 30 s, (annealing temperature listed in Table 2) for 30 s, 68 °C for 1 min; followed by a final extension cycle of 68 °C for 5 min.

**Table 2:** Primer sets used in this study

Primer name	Sequence (5′–3′)	Product size (bp)	Annealing temperature (°C)
PVL1 Forward	TCACACCTTGGGCATTCTCC	679	51
PVL1 Reverse	AAACCGAGACACCATCACCC		
PVL2 Forward	CTACTGACATGCGAGTTCC	185	45
PVL2 Reverse	CAGTCGACGAGACACAACCG		
PVL3 Forward	GATTTTGGTCATGGCAGC	435	45
PVL3 Reverse	CACTTGGCATACAGCTGG		
Rhabdo-like F	CCTTAGGCCGACGTCTTATCC	263	51
Rhabdo-like R	GAGCAGTTCTTTGGCTTGCC		

### MAVRIC ELISA

BHK cells infected with SREV, GGYV and WNV_KUN_ in 96-well plates were fixed with 200 µl per well 20% acetone fixative buffer [20% *v/v* acetone, 0.02% *w/v* bovine serum albumin in phosphate buffered saline (PBS, 140 mM NaCl, 2.7 mM KCl, 6 mM Na_2_HPO_4_, 0.9 mM KH_2_PO_4_)] overnight at 4 °C, or 150 µl/well formaldehyde fixative buffer (4% formaldehyde, 0.5% *v/v* Triton X-100 in PBS) for 10 min at 4 °C. After incubation, the fixative was removed, and plates were dried at room temperature for 48 h before use. BSR, OK and ISE6 cells inoculated with *I. holocyclus* homogenates were fixed using formaldehyde fixative buffer only.

Plates were pre-blocked with 150 µl/well TENTC blocking buffer (2% *w/v* casein, 0.05% *v/v* Tween-20, 10 mM Tris, 0.2 M NaCl, 1 mM EDTA, pH 8.0) for 1 h at room temperature. A cocktail of monoclonal antibodies (mAbs) 3G1 and 2G4 (MAVRIC) was prepared by diluting hybridoma supernatant in TENTC blocking buffer to previously determined optimal concentrations. Blocked plates were incubated with 50 µl/well MAVRIC for 1 h at 37 °C, followed by four washes with PBS containing 0.05% *v/v* Tween-20 (PBS-T). A 1:2000 dilution of secondary goat anti-mouse HRP (DAKO) in TENTC blocking buffer (50 µl/well) was then added, followed by another incubation for 1 h at 37 °C. Plates were washed six times with PBS-T and developed using 100 µl/well of substrate buffer (1 mM 2,2*-Azino-bis(3-ethylbenzothiazoline-6-sulfonic acid) (ABTS), 2.5 mM H_2_O_2_, in a solution prepared by mixing 0.1 M citric acid with 0.2 M Na_2_HPO_4_ to give a pH of 4.2) for 1 h at room temperature, protected from light. Absorbance was measured at 405 nm and samples with an average OD_405nm_ equal to or greater than twice that of the average mock reading were considered positive.

### Antigenic analyses

Antigenic analysis of SREV and GGYV was performed on BHK cells infected at a MOI of 0.1 for 48 h in 96-well plates and fixed with 20% acetone fixative buffer as described above. Serial two-fold dilutions of mAbs in TENTC blocking buffer were tested. For mAbs in hybridoma supernatant a starting dilution of neat (P3H8) or 1:5 (4G2, 6B6C) was used, while 1D1 (purified mAb) and M2-1E7 (ascitic fluid) were used at a starting dilution of 1:500. Primary antibody was added to plates at 50 µl/well and incubated at 37 °C for 1 h. Fifty µl of goat anti-mouse HRP (1:2000) was added to plates and incubated for 30 min at 37 °C.

Anti-FCV ELISA using mouse immune sera was performed on BSR cells infected with FCV or TAGV at MOI of 0.1 for 5 days and fixed with acetone fixative buffer as described above. Serial dilutions of anti-FCV serum of 1:200 to 1:25,600 were prepared in TENTC blocking buffer. Both primary and secondary antibodies (goat anti-mouse 1:2000, DAKO) were added to plates at a volume of 50 µl/well and incubated for 1 h at 37 °C.

All plates were pre-blocked, washed and developed as described for the MAVRIC ELISA.

### Purification of viruses

Partial purification was performed on CMCV and FCV. Five T175 flasks per virus were seeded with BSR cells at 80% confluence 1 day prior to infection. Cells were infected with CMCV or FCV at a MOI of 0.1 for 1 h at room temperature with gentle agitation. Inoculum was removed and replaced with 18 ml 2% FBS/DMEM per flask. Cells were incubated for 5 days at 37 °C before harvesting supernatant and clarifying by centrifugation at 3000 *g* for 15 min at 4 °C. Two parts clarified supernatant was mixed with one part 40% PEG6000 solution (40% w/v polyethylene glycol 6000 in PBS) overnight at 4 °C. Virus was pelleted by centrifugation at 21,612*g* for 1 h at 4 °C in a JLA16.250 Beckman-Coulter rotor. Precipitated virus was resuspended in cold PBS. As the pathogenicity of CMCV and FCV in mice is unknown, the virus preparations were inactivated in preparation for their use as antigens for immunisation of mice. To do this a solution of binary ethyleneimine (BEI) was prepared by the addition of 3–10 mM 2-bromoethylamine to DMEM brought to pH 9.0 with 2 N NaOH and warmed to 37 °C. PEG-precipitated virus was resuspended in the BEI-DMEM solution and incubated at 37 °C overnight before BEI was neutralised by the addition of sodium thiosulfate to a final concentration of 0.1 M. The inactivated virus preparation was then layered onto a cushion of 20% sucrose in NTE buffer (10 mM Tris, 1 mM EDTA, 120 mM NaCl, pH 8.0) followed by ultracentrifugation at 133,668*g* for 2 h at 4 °C in a SW32 Ti rotor (Beckman-Coulter). Pelleted virus was incubated in PBS at 4 °C overnight. After pellets were resuspended, the samples underwent three rounds of buffer exchange in 5 ml PBS using high molecular weight (100 K) MWCO Spin-X UF concentrator columns (Corning) before concentrating to a final volume of 100–200 µl. Purified virus was stored at − 80 °C until use.

### Protein analysis by SDS-PAGE and western blot

Purified virus preparations were diluted in 4 × NuPAGE LDS sample buffer (Invitrogen) without boiling or reducing and analysed by SDS-PAGE using a 4–12% Bis–Tris gel (NuPAGE) at 170 V for 42 min in 1 × Tris-glycine running buffer. Gels were stained using SYPRO Ruby protein stain (Thermo Fisher Scientific) as per manufacturer’s instructions and imaged on a UV transilluminator.

FCV or uninfected cell lysate was prepared by harvesting BSR cells from a T75 flask in NP-40 lysis buffer (1% NP-40 in 150 mM NaCl, 50 mM Tris-HCl [pH 7.3], 1:100 protease inhibitor cocktail (P8340, Sigma, St Louis, MO, USA)) for 30 min at 4 °C. Lysate was clarified by centrifugation at 10,000 *g* for 10 min at 4 °C and stored at − 80 °C until use. To test anti-FCV immune sera, electrophoresis of cell lysates was performed as described above and proteins were transferred to a nitrocellulose membrane at 30 V for 60 min. The membrane was pre-blocked with TENTC blocking buffer and probed with anti-FCV serum diluted 1:200 in TENTC blocking buffer. The membrane was washed three times with Tris-buffered saline (20 mM Tris-HCl pH 7.5, NaCl: 150 mM, 0.05% *v/v* Tween-20 detergent) before addition of goat-anti mouse conjugated with IRDye800 (LI-COR) diluted at 1:8000 in blocking buffer for 1 h at room temperature. The blot was imaged using the Odyssey CLx.

### Mass spectrometry

FCV protein bands, which were visible by eye after gel electrophoresis, were excised under UV illumination and stored at − 20 °C. Gel slices were destained overnight at 37 °C in a solution of 50% acetonitrile/50 mM ammonium acetate. Proteins were reduced with 10 mM dithiothreitol for 1 h and cysteine residues were alkylated by addition of 25 mM acrylamide and incubated for 1 h at 23 °C. Gel slices were dried and peptides were digested overnight with 500 ng trypsin in a solution containing 50 mM ammonium acetate solution. Digested peptides were desalted and analysed using liquid chromatography mass spectrometry (LC-MS/MS) as described in [37]. Peptides were identified using ProteinPilot Software 5.0.2.0 (SCIEX) and a custom protein database containing *Mesocricetus auratus* (BSR cell line) proteins and the amino acid sequences of FCV RdRp, nucleocapsid and glycoprotein precursor. Standard search settings included: sample type, identification; digestion, trypsin; Cys alkylation, acrylamide; search effort, thorough; ID focus, biological modifications. False discovery rate analysis using ProteinPilot was performed on all searches.

### Generation of mouse immune serum to Finch Creek virus

All animal procedures had received prior approval from The University of Queensland Animal Ethics Committee (AEC #SCMB/329/15/ARC) and, where necessary, were performed under ketamine:xylazine anaesthesia. BEI-inactivated FCV along with inulin-based adjuvant Advax (Vaxine Ltd., Adelaide, Australia) was used to immunise a 6-week-old BALB/c mouse (Animal Resources Centre, Murdoch, Western Australia, Australia) via the subcutaneous route (s.c.). Mice were kept on clean bedding and given food and water ad libitum. The immunized mouse was bled via the tail vein at 2 weeks following immunization and the serum tested for evidence of seroconversion to FCV using a fixed-cell ELISA as described above. A second dose of antigen was delivered 6 weeks after the initial dose. Two weeks after receiving the second dose, the mouse was killed by a ketamine:xylazine overdose and blood for serum-procurement was harvested via cardiac puncture.

### Sequencing of viruses

Nugget virus (NUGV, MI14847) RNA was extracted from culture supernatant from an archival virus stock using the QIAamp Viral RNA extraction kit (QIAGEN, Hilden, Germany) with carrier RNA omitted and DNA Wipeout was used to remove residual cell line DNA (Qiagen). First-strand cDNA synthesis was performed with Protoscript II kit using the supplied random primer mix (New England Biolabs; Ipswich, MA, USA). Second-strand DNA synthesis was performed using the NEBNext mRNA Second-Strand Synthesis Module (New England Biolabs). The Nextera XT library kit (Illumina; San Diego, CA, USA) was used to construct cDNA libraries with individual samples barcoded. All reactions were performed according to the conditions recommended by the respective manufacturers. Library sequencing was performed by the Australian Genome Research Facility (AGRF) on a HiSeq Illumina sequencer (125 nt paired end reads). Sequence assembly was performed using the de novo assembly tool in Geneious v8 at low sensitivity using 125 bp read lengths.

Preparations of FCV EB06 and CMCV I-2 for sequencing were generated by PEG-precipitation of virus from supernatant of 2 × T175 flasks of BSRs as described above. Sequencing for these viruses was performed as described for NUGV with the following modifications; DNAse treatment was performed using Heat&Run DNase (ArcticZymes); second-strand DNA synthesis was performed using *Escherichia coli* DNA ligase, DNA polymerase I and ribonuclease H (New England Biolabs). The resulting libraries were sequenced on a NextSeq 500 generating 2 × 151 base pair paired reads. The viral genome was assembled using Geneious R8 software as described above.

### Growth kinetics

ISE6 and BSR cells were seeded in 24-well plates at a density of 10^5^ cells per well, 24 h prior to infection. For NUGV, one replicate from each time point was seeded onto sterile glass coverslips in 24-well plates. On the day of infection, seeding media was removed and virus inoculum prepared in complete ISE6 cell media (10% FBS, 10% tryptose phosphate broth, 0.1% bovine lipoprotein solution, pH 7.5) or 2% FBS/DMEM was added to cells at a MOI of 0.1. Inoculated cells were incubated at room temperature with gentle rocking for 1 h before inoculum was removed and monolayers were washed three times with 1 ml/well sterile PBS. Supernatant from triplicate wells was harvested at 2, 12, 24, 48, 72, 96, 120 and 168 h post-infection (hpi) and stored at − 80 °C. For NUGV, supernatant was also harvested at 144 hpi and coverslips were harvested at each time point and fixed with 1 ml of formaldehyde fixative buffer (4% formaldehyde, 0.5% *v/v* Triton X-100 in PBS) at 4 °C for 10 min and air dried for at least 48 h before storing at − 20 °C.

Quantification of viral titres in supernatants collected at each time point was determined using a modified TCID_50_ method described in [27, 38]. Briefly, ten-fold dilutions of supernatant (undiluted to 1:10^8^) were prepared in 2% FBS/DMEM. Each dilution (50 µl) was added to four replicate wells of a 96-well plate and incubated for 5 days at 37 °C with 5% CO_2_. Flavivirus (GGYV, SREV, WNV_KUN_ NSW2011 strain), NUGV and FCV supernatant titrations were performed on BSR cells, while CMCV titrations were performed on Vero cells. After 5 days, plates containing flaviviruses and FCV were fixed using acetone fixative buffer and virus replication was determined by fixed-cell ELISA using anti-flavivirus E mAb 4G2 or anti-FCV immune serum as described above. NUGV-infected cells were fixed with 150 µl/well formaldehyde fixative buffer for 10 min at 4 °C and virus replication was determined by MAVRIC ELISA as described previously [27]. For CMCV, virus replication was scored by the presence or absence of CPE. Viral titres in each sample were then calculated using the Reed-Muench guidelines [36].

### Immunofluorescence assays

ISE6 cells were seeded on glass coverslips in 24-well plates at 10^5^ cells per well 1 day prior to infection. Cells were infected at MOI 0.1 for 1 h at room temperature before inoculum was removed and replaced with 1 ml complete ISE6 medium. Cells were incubated at 34 °C without CO_2_. At 7, 14 and 28 dpi supernatants were harvested and stored at − 80 °C and coverslips were fixed by submerging in 100% ice-cold acetone for 5 min before air drying and storing at − 20 °C. Viral titres in supernatants were determined as described in the previous section. The protocol for E and dsRNA dual-labelling is as follows. All antibodies were diluted in TENTC blocking buffer to previously determined optimal concentrations before use. Coverslips were blocked with 900 µl of TENTC blocking buffer for 1 h at room temperature, followed by incubation with 300 µl anti-E mAb 4G2 at 28 °C for 1 h. Coverslips were washed thrice with 1 ml PBS-T (PBS with 0.05% *v/v* Tween-20) and incubated with 150 µl 1:500 Alexa Fluor 488-conjugated goat anti-mouse in TENTC blocking buffer for 1 h at 28 °C. Another 3 × PBS-T washes were performed followed by addition of 300 µl/well MAVRIC hybridoma supernatant (a cocktail of mAbs 3G1 and 2G4) and incubation at 37 °C for 1 h. Coverslips were again washed three times and Alexa Fluor 594-conjugated goat anti-mouse IgM (µ chain) diluted 1:500 in blocking buffer was added at 150 µl/well at 28 °C for 1 h. Secondary anti-IgM antibody solution was then removed and nuclear staining was performed with 150 µl/well Hoechst 33342 for 5 min at room temperature, protected from light. Three final PBS-T washes were performed, followed by one wash with PBS (without Tween-20), and coverslips were mounted on glass microscope slides using ProLong Gold Antifade mountant (ThermoFisher, Carlsbad, CA, USA). Images were taken on a Zeiss EPI fluorescence microscope.

For comparison of MAVRIC and J2 dsRNA immunolabelling, BSR cells were infected with GGYV, SREV or WNV_NSW2011_ at MOI 0.1 for 5 days and fixed with 1 ml per well of 4% formaldehyde in PBS at 4 °C for 10 min and air dried for at least 48 h before storing at − 20 °C. Prior to labelling, coverslips were thawed and permeabilised by incubation in 1 ml of 0.5% Triton X-114 *v/v* or 0.5% *w/v* digitonin in PBS for 10 min at 4 °C. For the anti-dsRNA time course, coverslips containing BSR cells infected with GGYV at MOI 0.1 were harvested at 12, 24, 48 and 96 hpi and fixed with 100% acetone as described above.

Immunolabelling for dsRNA was performed on pre-blocked coverslips with 300 µl of MAVRIC hybridoma supernatant or 150 µl of purified IgG mAb J2 diluted 1:200 in blocking buffer for 1 h at 37 °C. Coverslips were washed as described above and incubated with 150 µl of Alexa Fluor 488-conjugated goat anti-mouse (H + L) diluted in 1:500 TENTC blocking buffer for 1 h at 37 °C. Nuclear staining and mounting were performed as described above. Coverslips were imaged using a Zeiss LSM 510 META confocal microscope.

### Sequence annotation and phylogenetic analysis

Pairwise alignments of the nucleotide and amino acid sequences of archival viruses were performed in Geneious Prime v2019 using MUSCLE or ClustalW algorithms respectively. Sequence annotation for CMCV and FCV was performed using the following platforms: protein topology, Phobius [39]; N- and O-linked glycosylation site prediction, NetNGlyc 1.0 and NetOGlyc 4.0 servers [40]; domain prediction, HMMER EBI tools web server [41]; cleavage site predictions, ProP 1.0 server [42].

Nucleotide coding sequences for NUGV and orbivirus VP1, VP7 (T13), T2 and VP5 (OC2) proteins were aligned using the MAFFT v7.471 tool via the CIPRES gateway [43, 44]. Alignments were trimmed to the length of each NUGV segment and JModelTest2 via the CIPRES gateway was used to determine the best available model for each gene [45]. Trees were constructed in MEGA7 using the maximum likelihood method based on the Jukes-Cantor model with complete deletion of gaps and missing data with the following models for rates among sites used; for VP1 JC + G with four discrete categories, for VP7 (T13), T2 and VP5 (OC2) JC + I. Phylogenetic tests were performed for each node using 1000 bootstrap replicates. Neighbour-Join and BioNJ algorithms were used to generate initial trees based on a matrix of pairwise distances estimated using the maximum composite likelihood (MCL) approach.

Virus-like contigs generated from *I. holocyclus* transcriptome data [46] were first assessed by a Blastx database search (database: nr, August 2021) and open reading frames were determined using the Translate Tool via the ExPASy website. Amino acid sequences for the nucleocapsid proteins of Phlebovirus-like sequence 1 (PVL1) and 3 (PVL3) were aligned with selected phlebovirus nucleocapsid proteins using the MAFFT alignment algorithm via the EBI-EMBL web interface [39]. Maximum-likelihood analysis was performed on a MAFFT alignment of 27 sequences trimmed to 338 amino acids before non-conserved regions were removed using GBlocks with less stringent settings (smaller final blocks, gap positions in final blocks and less stringent flanking positions allowed). A final alignment of 99 positions was used for phylogenetic analysis in MEGA7 using the LG model with 1000 bootstrap replicates. Gamma distribution was used to model evolutionary rate differences among sites with some sites allowed to be evolutionarily invariable (LG + G + I). Neighbour-Join and BioNJ algorithms were applied to a matrix of pairwise distances estimated using the Jones Taylor Thornton (JTT) method to generate the initial trees and topology with superior log likelihood value was selected automatically.

The protein structures of PVL1 and PVL3 nucleocapsid proteins were predicted using the Phyre2 Protein Fold Recognition Server based on an alignment with Toscana virus N protein with 100% confidence [47]. The 3D protein model figures were produced using Chimera software version 1.13.1 [48] and regions were annotated based on domains identified in [49, 50].

## Results

### Growth and antigenic characterisation of Australian tick-borne flaviviruses

The mosquito-borne flavivirus West Nile virus subtype Kunjin (WNV_KUN_) is routinely used as a positive control for double-stranded RNA production in the MAVRIC system [23]. Therefore, for adapting the assay for discovery of tick viruses, we assessed two tick-borne flaviviruses GGYV and SREV for this purpose. Initially we compared the reactivity of a panel of six pan-flavivirus anti-E mAbs against WNV_KUN_-, GGYV- and SREV-infected BHK cells in fixed-cell ELISA. Five of the six mAbs tested reacted to all three flaviviruses (Table 3). However, we found that the anti-E reactive mAb M2-1E7 bound to GGYV but not SREV (Table 3). Neither GGYV or SREV was detected in infected BHK cells by MAVRIC ELISA after fixation with 20% acetone fixative buffer or 4% formaldehyde with 0.5% Triton X-100, despite detecting WNV_KUN_ under both conditions (Table 3).

**Table 3:** Reactivity of pan-flavivirus mAbs and MAVRIC against SREV and GGYV in fixed-cell ELISA

Antibody	Reactive protein	Reactivity in fixed-cell ELISA^a^			References
		WNV_KUN_	GGYV	SREV	
4G2	Envelope	+ + +	+ + +	+ +	[71]
M2-1E7	Envelope	+ + +	+ +	−	[72]
6B6C	Envelope	+ + +	+ +	+	[73]
3H6	Envelope	+ + +	+ +	+	[74]
1D1	Envelope	+ + +	+ +	+	[75]^b^
P3H8	Envelope	+ + +	+	+	[76]
MAVRIC	dsRNA	+ +	−^c^	−^c^	[23]

^a^+ + + , OD_405nm_  ≥ 2 for all tested dilutions; + + , OD_405nm_ ≥ 1 for all tested dilutions; +, OD_405nm_ < 1 for all tested dilutions; −, OD_405nm_ ≤ mock OD for all tested dilutions

^b^mAb prepared by A.K. Falconar, London School of Hygiene and Tropical Medicine, London, UK

^c^Greater than mock OD but beneath the threshold for a positive score (2 × mock OD_405nm_). Negative for MAVRIC in fixed cell ELISA using both 20% acetone and 4% formaldehyde with 0.5% Triton X-100 fixation

**Fig. 2:**
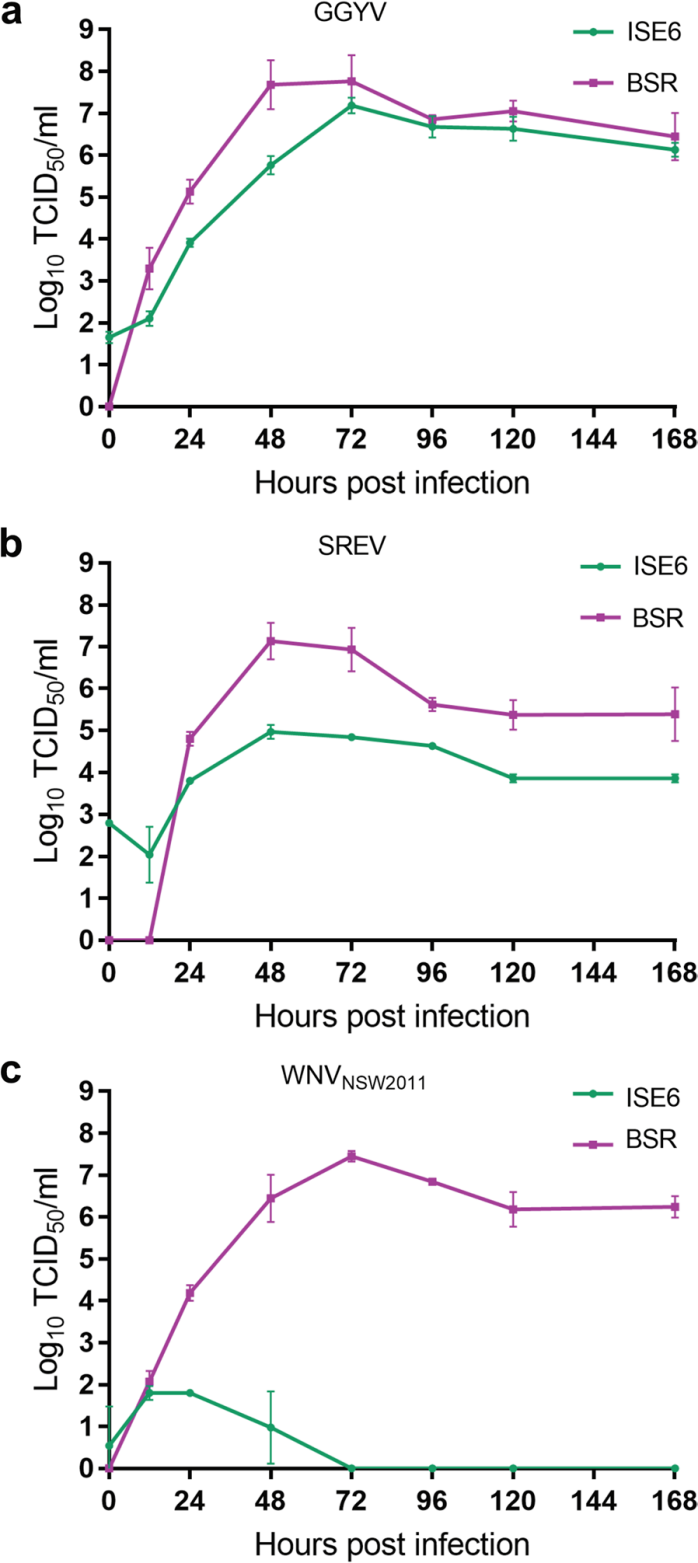
In vitro growth kinetics of tick-borne flaviviruses **a** GGYV and **b** SREV and **c** mosquito-borne flavivirus WNV_KUN_ strain NSW2011 in vertebrate (BSR) and tick (ISE6) cells. Viral titres in supernatants from three replicate wells were determined by TCID_50_ method after titration on BSR cells

To understand the growth dynamics of tick-borne flaviviruses compared to their mosquito-borne relatives, a growth kinetics assay was performed on WNV_KUN_, GGYV and SREV in vertebrate and tick cells over 7 days. As our preliminary data indicated that prototype WNV_KUN_ strain (MRM61C) did not replicate in ISE6 cells, we tested for replication of the more neuroinvasive WNV_KUN_ strain NSW2011 [51]. This assay revealed a similar growth pattern for all three flaviviruses in BSR cells. However, both tick-borne viruses reached peak titre at 48 hpi, 24 h earlier than the mosquito-borne WNV_KUN_ (Fig. 2). GGYV replicated robustly in ISE6 cells, reaching an average peak titre of 10^7.19^/ml at 72 hpi, which was within 1 log of the titre recovered from vertebrate cells at this time point (average titre 10^7.77^/ml) (Fig. 2a). While the growth curve of SREV in ISE6 cells mirrored that of the virus in BSRs, viral titres recovered from the tick cells were lower than those recovered from BSR cells at all time points, with > 2 log difference between the peak titres recovered from each cell line (ISE6: 10^4.97^/ml, BSR 10^7.13^/ml) (Fig. 2b). No infectious virus could be detected in supernatant from WNV_KUN_-infected tick cells from 72 hpi onwards. However, a small amount of virus (average titre 10^1.8^/ml) was detected in supernatant harvested from three replicate wells at both 12 and 24 hpi followed by a decline at 48 hpi, indicating that some viral replication may occur in these cells during the early stages of infection (Fig. 2c).

### Detection of double-stranded RNA in tick-borne flavivirus replication

**Fig. 3:**
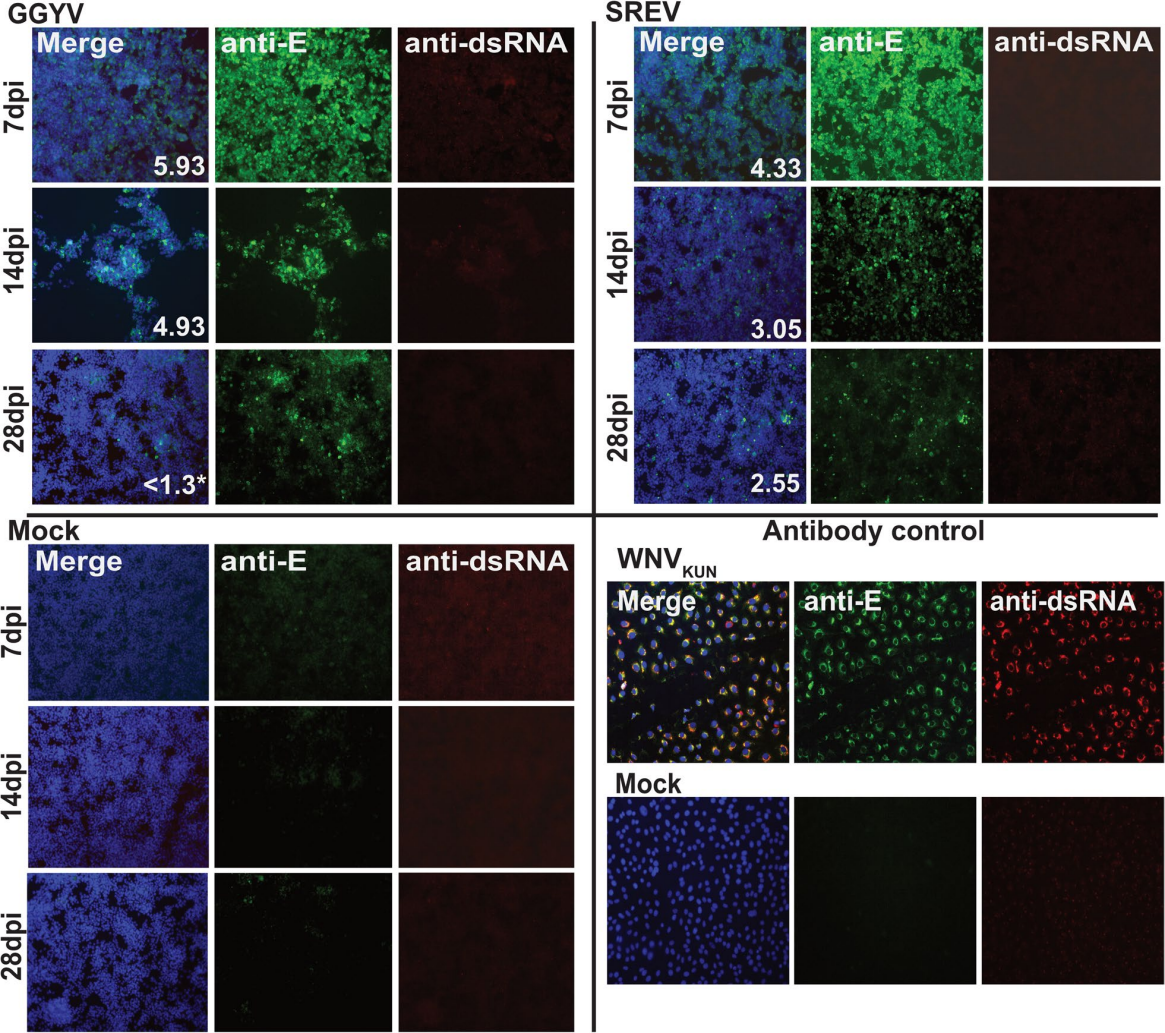
Immunofluorescence assay (IFA) on 100% acetone-fixed ISE6 cells infected with GGYV and SREV at 7, 14 and 28 days post-infection (dpi). Immunofluorescent labelling with anti-E mAb 4G2 (green), anti-dsRNA MAVRIC (red) and Hoechst nuclear stain (blue). Mock controls, uninfected cells inoculated with culture medium only. WNV_KUN_-infected Vero cells were used as assay controls. The log value of average virus titres (TCID_50_ units/ml) recovered from supernatant harvested at each time point is indicated in white text at the bottom right corner of each merged panel. *Titre below the limit of detection (< 10^1.3^/ml). Images taken at 20 × magnification with an EPI fluorescence microscope

**Fig. 4:**
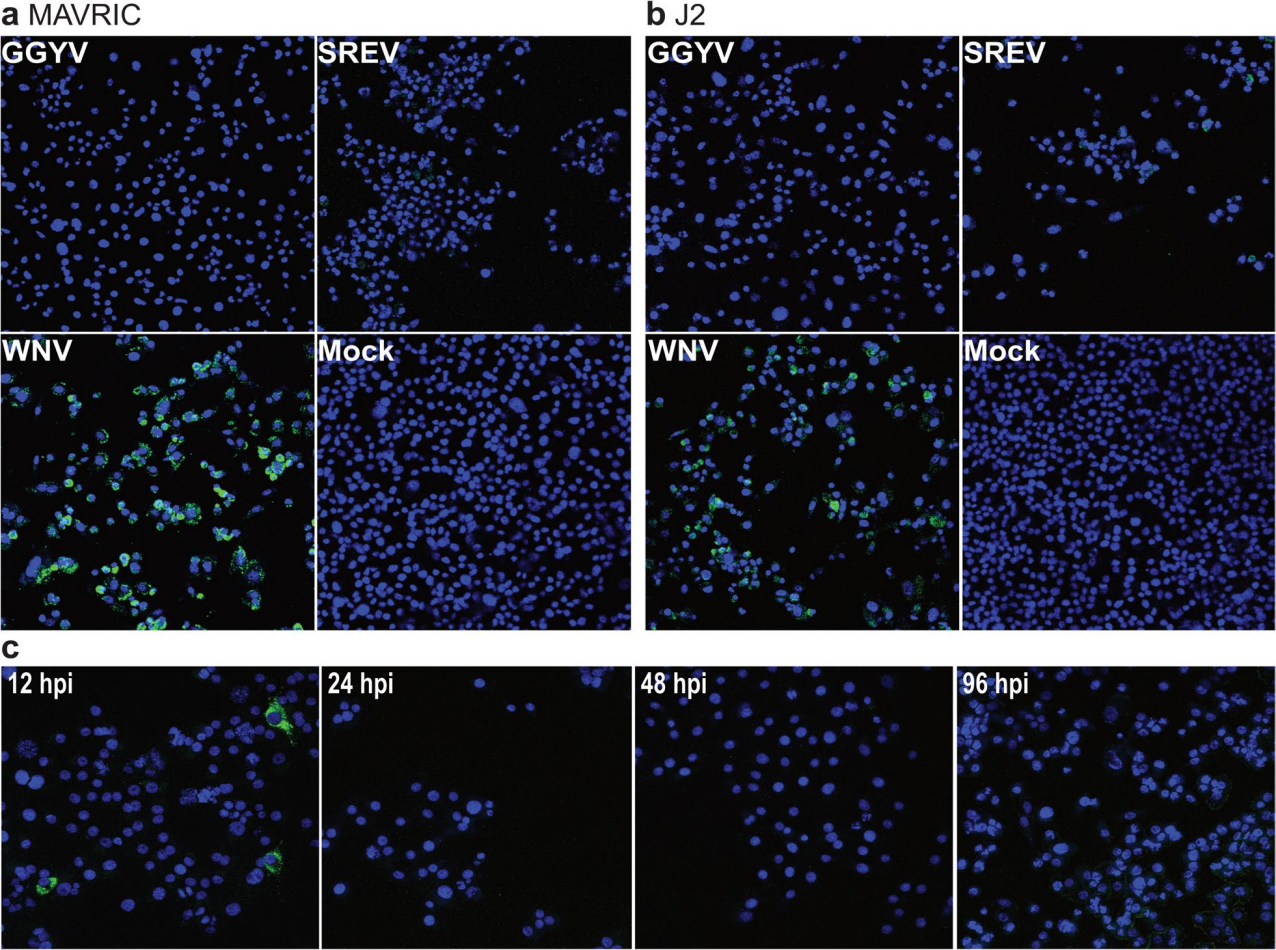
IFA analysis of tick- and mosquito-borne flavivirus-infected cells using anti-dsRNA mAbs **a** MAVRIC and **b** J2. Representative images of BSR cells infected with GGYV, SREV, WNV_KUN_ or mock-infected. At 5 dpi cells were fixed with 4% formaldehyde and permeabilised with 0.5% Triton X-114. **c** GGYV-infected BSR cells fixed with 100% acetone and immunolabelled for dsRNA using mAb J2 at early time points post-infection. All images were taken at 20 × magnification. Blue, nuclei; green, dsRNA

While our growth kinetics data indicated that GGYV and SREV replicated in both tick and vertebrate cell lines, we were unable to detect dsRNA in cells infected with either virus using the MAVRIC ELISA. To investigate this further we performed an immunofluorescence assay (IFA) on ISE6 cells infected with GGYV or SREV. Infectious virus was detected in supernatant from cells infected with SREV at 7, 14 and 28 dpi, but only from GGYV-infected cells at 7 and 14 dpi (Fig. 3). Envelope protein could be detected by IFA for both viruses at all time points; however, no dsRNA was detected in these cells by co-labelling with MAVRIC (Fig. 3). To confirm that the lack of dsRNA immunolabelling was not related to our anti-dsRNA antibodies, we compared detection of dsRNA in SREV-, GGYV- and WNV_KUN_-infected cells using MAVRIC (mAbs 3G1 and 2G4) and the commercial anti-dsRNA antibody J2 (IgG, Scicons) [52]. While both antibodies detected dsRNA in WNV_KUN_-infected BSR cells at 5 dpi, neither J2 nor MAVRIC detected significant amounts of dsRNA in SREV- and GGYV-infected cells at this time point following formaldehyde fixation and permeabilisation with 0.5% Triton X-114 (Fig. 4a, b). This result was also observed in cells fixed and permeabilised using 4% formaldehyde with 0.5% digitonin (Additional file 1: Fig. S1).

As we were unable to detect dsRNA by IFA or ELISA at 5 dpi onwards, we performed anti-dsRNA labelling on GGYV-infected BSRs at earlier time points. This analysis showed that dsRNA was not detected using J2 at 24, 48 and 96 hpi (Fig. 4c). However, a small percentage of cells with perinuclear dsRNA signal were observed at 12 hpi (Fig. 4c).

### Growth kinetics and in situ detection of NUGV in vertebrate and tick cells

NUGV is an orbivirus that was isolated from *I. uriae* ticks collected at Macquarie Island [9]. We first assessed its ability to replicate and produce sufficient levels of accessible viral dsRNA in inoculated BSR and ISE6 cells for detection by MAVRIC.

**Fig. 5:**
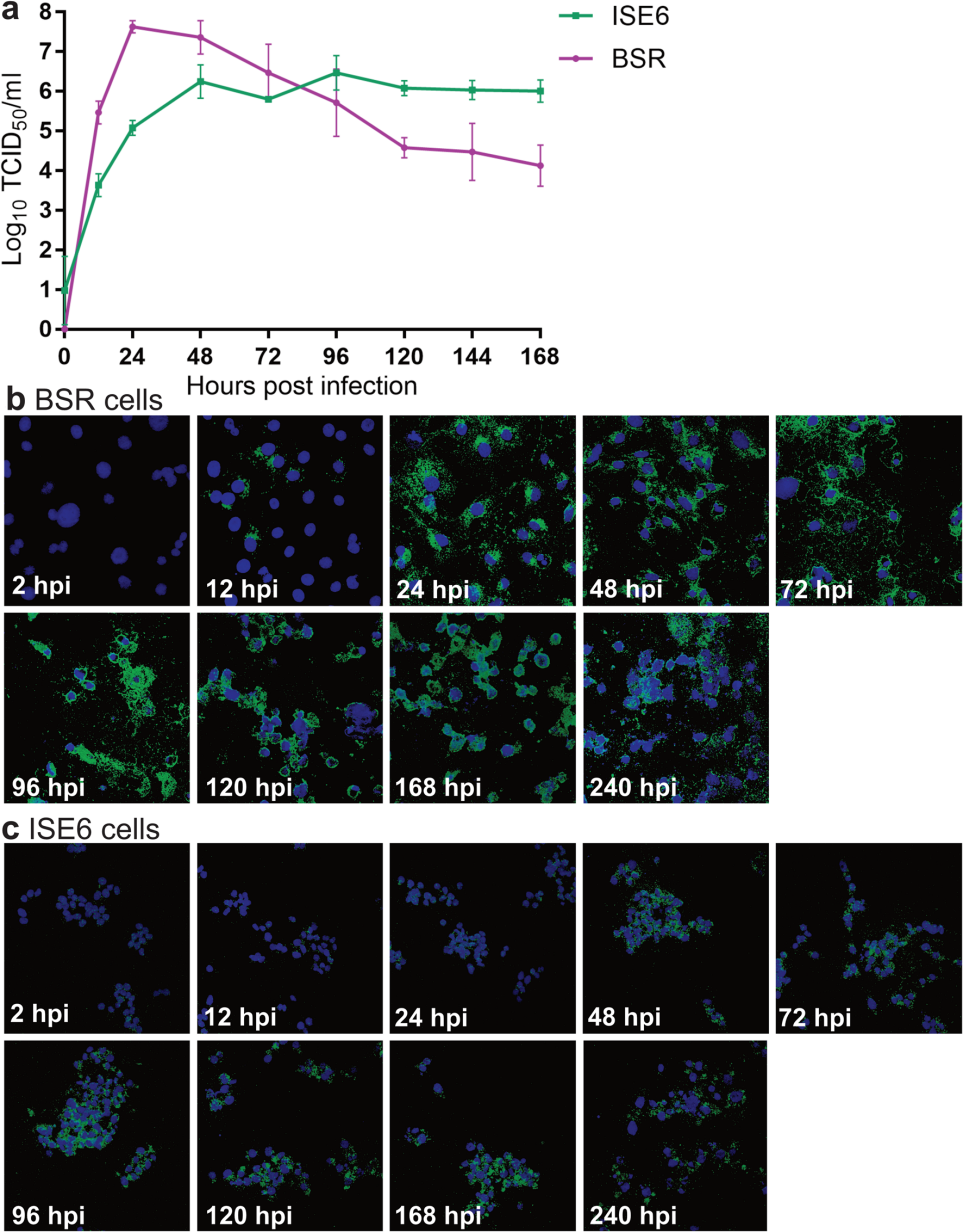
In vitro replication of NUGV in vertebrate and tick cell lines. **a** Growth kinetics analysis of NUGV in ISE6 and BSR cells. Viral titres in supernatants from three replicate wells were determined by TCID_50_ method after titration on BSR cells. **b** Detection of dsRNA in NUGV-infected BSRs at various time points post-infection. **c** Detection of dsRNA in NUGV-infected ISE6 cells at various time points post-infection. Blue, Hoechst nuclear stain; green, anti-dsRNA immunolabelling by MAVRIC. Images taken at 40 × magnification

Growth kinetics analysis showed NUGV replicated to an average peak titre of 10^7.62^/ml by 24 hpi in BSR cells. This was followed by a steady decline in titre over the next 6 days (Fig. 5a), which coincided with overt cytopathic effect in these cells. In ISE6 cells, an average peak titre of 10^6.47^/ml was detected at 96 hpi, with titres staying relatively stable from this point on (Fig. 5a). Infected cells were fixed at each time point and immunolabelled for dsRNA using MAVRIC. Significant amounts of dsRNA were first detected in BSR cells at 24 hpi, coinciding with the peak titre of the virus (Fig. 5b). In ISE6 cells, a strong dsRNA signal was detected from 48 hpi onward (Fig. 5c).

### Genomic sequence analysis of NUGV

**Fig. 6:**
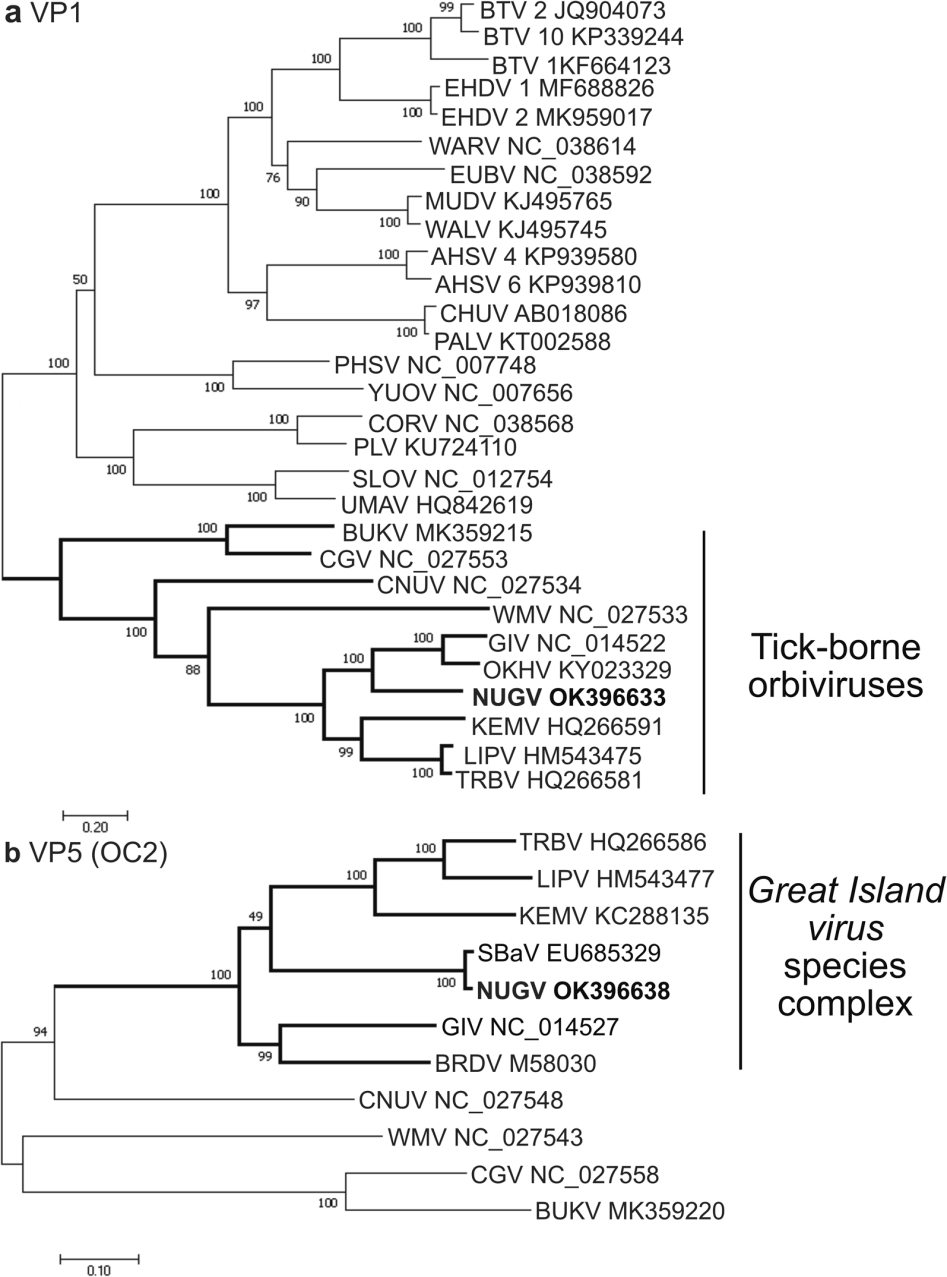
Phylogenetic relationship of NUGV and members of the genus Orbivirus. Phylogenetic trees were constructed from the aligned nucleotide coding sequences of **a** VP1 (polymerase) and **b** VP5 (OC2) for tick-borne orbiviruses only. Phylogenetic analysis of VP1 sequences was performed in MEGA7 using the JC + G = 4 model. Bold branches denote members of the tick-borne orbivirus group. VP5 phylogenetic analysis was performed in MEGA7 using the JC + I model and bold branches denote the *Great Island virus* species complex. The percentage of trees in which the associated taxa clustered together is shown next to the branches

Although NUGV was first isolated in 1972 [9], to date no sequence analysis has been conducted on the viral genome. We performed deep sequencing on an archival stock of the prototype NUGV strain (MI14847) and obtained partial sequence for all ten genome segments (Table 4). Pairwise alignments with available sequence for SBaV, an orbivirus isolated from *I. uriae* collected on Macquarie Island in 2002 [15], showed high similarity at both the nucleotide (96.4–98.4%) and amino acid (97–100%) levels for VP4 (CaP), VP5 (OC2), NS2 (ViP) and NS3 (Table 4). NUGV also shares approximately 75% nucleotide and 90–91% amino acid identity with the T2 and T13 sequences of Great Island virus (GIV) (Table 4). Phylogenetic analysis of the nucleotide sequences of VP1 genes shows that NUGV groups with GIV within the tick-borne orbivirus clade (Fig. 6a). This grouping was also supported by phylogenetic trees constructed from the nucleotide sequences of the T2 and VP7 (T13) structural genes (Additional file 1: Fig. S2). Maximum likelihood analysis of the nucleotide sequence of VP5 (OC2) shows that NUGV and SBaV cluster with other members of the GIV species (Fig. 6b).

**Table 4:** Summary of sequence data generated for Nugget virus

Expected segment^a^	Gene^b^	Accession number	Segment length (nt)	Protein length (aa)	% Pairwise identity with GIV (nt/aa)^c^	% Pairwise identity with SBaV (nt/aa)^d^
1	VP1 (Pol)	OK396633	3776	1258	69.4/80.3	–
2	VP3 (T2)	OK396634	2559	852	74.9/90.4	–
3	VP4 (CaP)	OK396635	1645	547	65.3/71.9	96.4/97.0
4	NS1 (TuP)	OK396636	1548	515	63.9/69.9	–
5	VP2 (OC1)	OK396637	1458	486	53.4/59.7	–
6	VP5 (OC2)	OK396638	1229	409	66.0/70.4	98.4/99.0
7	VP7 (T13)	OK396639	988	329	75.6/91.2	–
8	NS2 (ViP)	OK396640	1143	372	68.3/76.1	98.0/99.2
9	VP6 (Hel)	OK396641	959	314	64.9/51.4	–
10	NS3	OK396642	566	157	66.9/72.0	98.0/100.0

^a^Segment predictions are based on GIV segment designations [77]

^b^Viral protein designations (VP) are based on Bluetongue virus nomenclature as per [78]. Pol, RNA-dependent RNA polymerase; T2, conserved major subcore protein with *T* = 2 symmetry; CaP, capping enzyme; TuP, tubule forming protein; OC1, outer capsid protein 1; OC2, outer capsid protein 2; T13, major core protein with *T* = 13 symmetry; ViP, viral inclusion body protein; Hel, ssRNA- and dsRNA-binding helicase; *NS*, non-structural protein

^c^GIV nucleotide sequences (NC_014522 to NC_014531), amino acid sequences (YP_003896058 to YP_003896068)

^d^SBaV nucleotide sequences (EU685329 to EU685333), amino acid sequences (ACD38335 to ACD38337 and ACD38339)

### Characterisation of CMCV

**Fig. 7:**
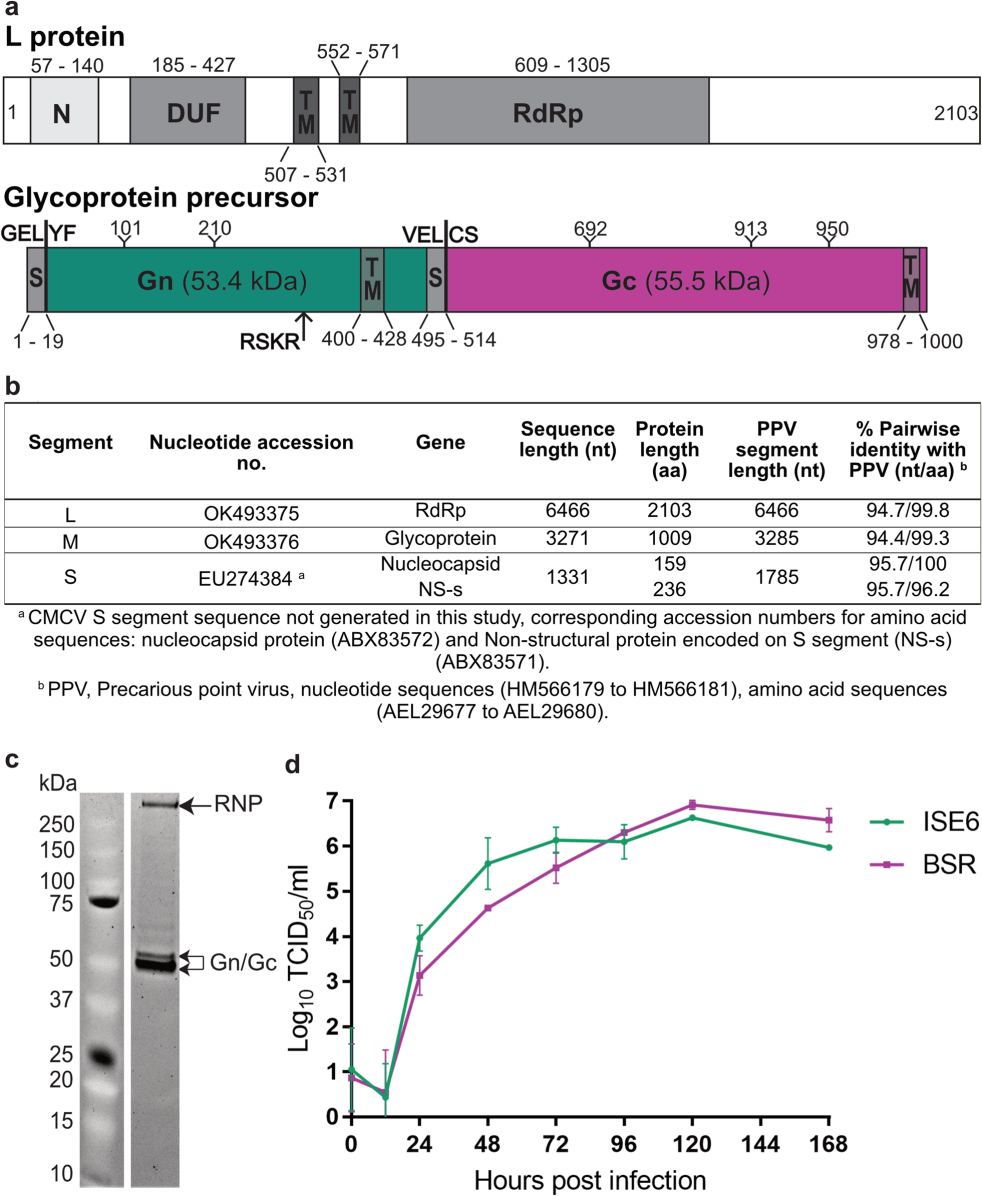
Sequence and in vitro analysis of the tick-borne phlebovirus CMCV. **a** Annotation of CMCV L protein and glycoprotein precursor. Numbers indicate relevant amino acid positions. Green: Gn protein. Magenta: Gc protein. The predicted molecular weight of each mature glycoprotein is indicated in brackets. Predicted signal peptidase cleavage sites preceding Gn (GEL-YF) and Gc (VEL-CS) are indicated by solid lines. An arrow indicates the position of a predicted furin cleavage site (RSKR) in the Gn protein. N, N-terminus; DUF, domain of unknown function (pfam: DUF3770); RdRp, RNA-dependent RNA polymerase; TM, transmembrane domain; S, signal peptide; Y, predicted N-glycan. **b** Summary of CMCV sequence data for the L and M segment generated in this study and similarity to Precarious point virus. CMCV S segment sequence was published in [15]. **c** SDS-PAGE analysis of purified CMCV. RNP, ribonucleoprotein complex; Gn/Gc, mature glycoproteins. **d** Growth kinetics of CMCV in vertebrate (BSR, baby hamster kidney) and tick (ISE6, *Ixodes scapularis*) cells. Viral titres in supernatants from three replicate wells were determined by TCID_50_ method after titration on Vero cells

We previously isolated CMCV from *I. uriae* ticks collected in 2002 from Macquarie Island [15] and reported that it phylogenetically clustered with other phleboviruses and was genetically similar to PPV, a virus previously isolated from the same tick species collected on the island almost 30 years earlier [10]. However, only the S segment of CMCV was originally sequenced. To complete the characterisation of this virus and its genetic relationships to other tick-borne viruses found in the same region, we performed next generation sequencing on a stock of CMCV. This yielded full-length L segment and near full-length sequence for the M segment (excluding the first 10 bases of the 5′ and last 4 bases of 3′ genomic termini). From this, the complete coding sequences (CDS) of the L protein and glycoprotein precursor were identified and annotated (Fig. 7a). The nucleotide sequences of the L and M segment shared 94.7% and 94.4% similarity with PPV, while the L protein and glycoprotein precursor shared > 99% amino acid similarity with this virus (Fig. 7b). The S segment of CMCV that had been generated previously shares 95.7% nucleotide similarity with the S segment of PPV and 100 and 96.2% amino acid similarity with PPV nucleocapsid and non-structural proteins respectively (Fig. 7b) [15].

Protein analyses of purified CMCV showed the Gn and Gc glycoproteins run at apparent molecular weights of 47.2 and 53.2 kDa (Fig. 7c). A third band was identified at 258.3 kDa, which may represent viral ribonucleoprotein complexes (RNP). Growth kinetics analysis showed that CMCV replicated to higher titres in ISE6 cells between 24 and 72 hpi compared to BSR cells (Fig. 7d). Peak titres were detected in both cell lines by 120 hpi (5 dpi) with the titres from BSR cells (10^6.91^/ml) surpassing that of ISE6 cells (10^6.63^/ml) (Fig. 7d).

### Genetic characterisation of FCV

**Fig. 8:**
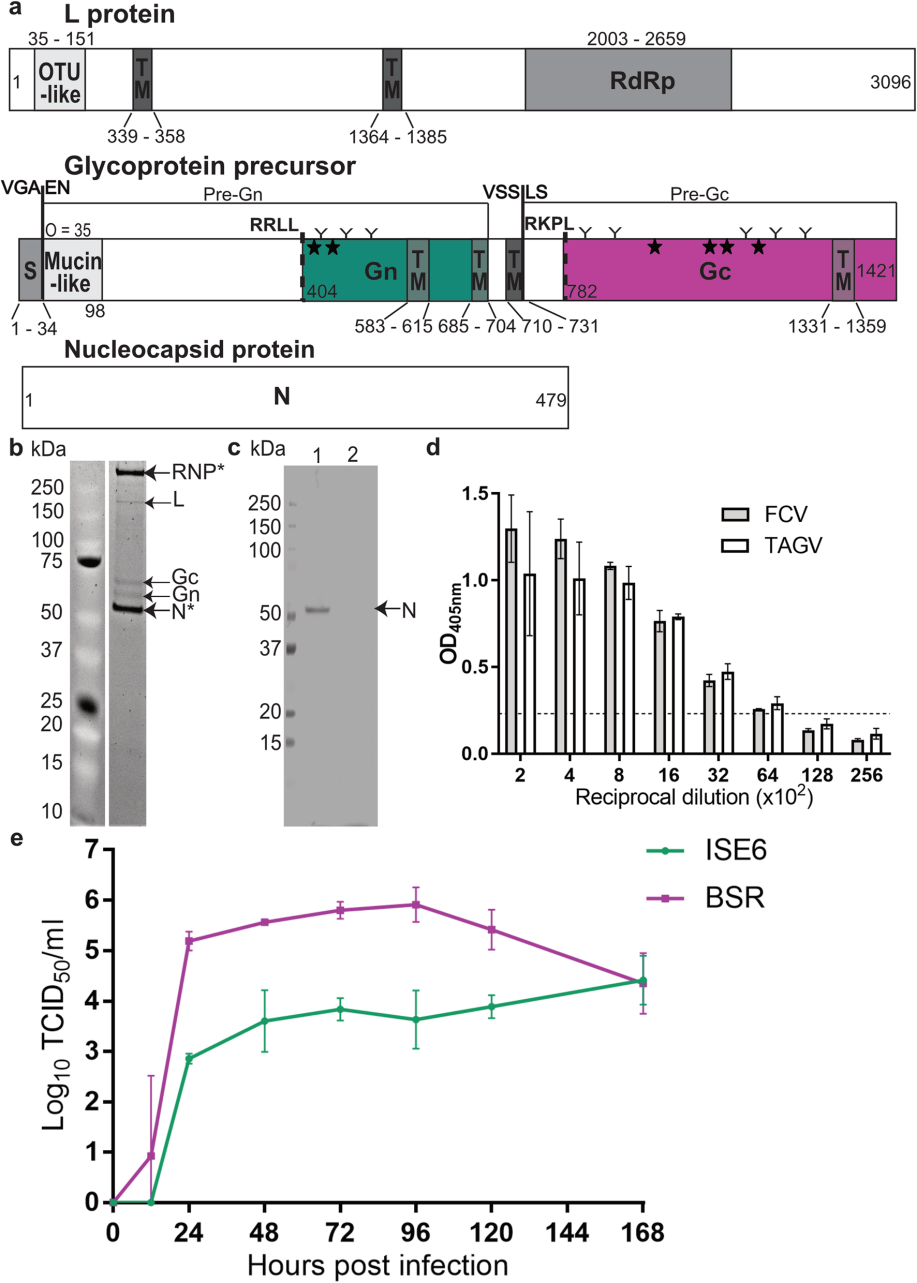
Finch creek virus sequencing and in vitro analysis. **a** Sequence annotation of FCV L, M and S polypeptides based on predictions of protein domains and cleavage sites. Numbers on the figure indicate relevant amino acid positions. OTU-like, ovarian tumor family-like domain. RdRp, RNA dependent RNA polymerase domain. Transmembrane domains (TM) are indicated by dark grey boxes. S, signal peptide. Signal peptidase cleavage sites for the Pre-Gn (VGA_34_EN) and Pre-Gc (VSS_733_LS) proteins are indicated by cleavage site sequence and bold solid line. Subtilisin kexin isozyme-1 (SKI-1) cleavage sites (RRLL, RKPL) are indicated by dashed lines. The number of predicted O-linked glycosylation sites in the mucin-like domain is indicated above the domain (O = number of sites). Green: Gn protein. Magenta: Gc protein. Predicted N- and O-linked glycans in the Gn and Gc proteins are indicated by Y or *, respectively. N, nucleocapsid protein. **b** SDS-PAGE of purified FCV virions. Presumed protein identities are indicated by arrows. Identities confirmed by mass spectrometry are denoted with *. **c** Western blot analysis of anti-FCV serum against lysate of FCV-infected (lane 1) or mock-infected (lane 2) cells. **d** Reactivity of anti-FCV mouse serum to FCV or TAGV-infected BSR cells in fixed cell ELISA. Values graphed represent average optical density (OD) reading at 405 nm after subtraction of mock-OD. Dotted line indicates the threshold of detection which is equal to 2 × the average OD value of mock-infected cells. **e** Growth kinetics of FCV in ISE6 and BSR cells. Viral titres in supernatants from three replicate wells were determined by TCID_50_ method after titration on BSR cells

We performed similar analyses on FCV, a nairovirus isolated from *I. uriae* also collected at Macquarie Island in 2002 [9]. Complete open reading frames (ORFs) for all three genome segments were generated by next generation sequencing and the resulting proteins annotated (Fig. 8a). The newly obtained L segment sequence shared 97.7% nucleotide and 99.2% amino acid similarity with the partial L segment sequence previously reported for this virus (Table 5) [15]. All three segments also shared high similarity at both the nucleotide (97.8–99.6%) and amino acid (99.6–100%) levels with the sequence of the prototype isolate of TAGV (MI14850), isolated from the same tick species collected on Macquarie Island in 1972 (Table 5) [9, 14]. FCV appears less closely related to a new variant of TAGV identified in *I. uriae* ticks collected in 2017 from Antarctica [53], with 81.4% nucleotide 94% amino acid sequence shared across the L segment, the most conserved of the three segments (Table 5).

**Table 5:** Summary of sequence data generated for Finch Creek virus

Segment^a^	Gene	Sequence length (nt)	Protein length (aa)	% Pairwise identity with TAGV MI14850 (nt/aa)^b^	% Pairwise identity with TAGV Paradise Bay (nt/aa)^c^	% Pairwise identity with FCV (nt/aa)^d^
L	RdRp	11,849	3906	99.4/99.9	81.4/94.08	97.7/99.2
M	Glycoprotein	4545	1421	97.8/99.6	73.8/79.7	–
S	Nucleocapsid	1603	479	99.6/100	79.8/89.4	–

^a^Accession numbers for FCV nucleotide sequences generated in this study L segment (OK493377), M segment (OK493378), S segment (OK493379)

^b^TAGV, Taggert virus prototype isolate MI14850 nucleotide sequences (KU925491 to KU925493), amino acid sequences (AMR73395 to AMR73397)

^c^TAGV, 2017 isolate from Paradise Bay, Antarctica. Nucleotide sequences (MT025170 to MT025172), amino acid sequences (QIS88061 to QIS88063)

^d^FCV original L segment sequence [15]. Nucleotide (EU267169) and amino acid (ABX80519)

### In vitro characterisation of FCV

We observed that after passaging FCV in ISE6 cells, the virus caused inconsistent CPE when inoculated onto mammalian cells and therefore could not be reliably quantified by CPE scoring. To circumvent this, we purified the virus for immunization of mice to generate virus-specific immune serum. As the pathogenicity of FCV in mice and humans is unknown, the virus was inactivated by the addition of binary ethylenimine (BEI) to cell culture supernatant prior to purification. SDS-PAGE analysis of the purified virions revealed two prominent bands at apparent molecular weights of 261.2 and 54.8 kDa (Fig. 8b). Both bands were determined by mass spectrometry to contain the nucleocapsid (N) protein (Fig. 8b, Additional file 2: Dataset S1). Three additional bands were identified at apparent molecular weights of 181.2, 72.2 and 62.2 kDa, which presumably contain the L protein, Gc and Gn glycoproteins respectively (Fig. 8b).

Mouse immune serum raised to this virus preparation was assessed in western blot and showed reactivity to the N protein in FCV-infected cell lysate (Fig. 8c). Titration of the immune serum on FCV and TAGV infected BSRs in fixed-cell ELISA showed the serum detected both viruses equally well up until an end point dilution of 1:6400 (Fig. 8d).

Growth kinetics analysis of FCV showed that the virus replicated rapidly in the first 24 h followed by a plateau in both vertebrate and tick cell lines (Fig. 8e). In BSR cells an average peak titre of 10^5.91^/mL was detected at 96 hpi followed by a decline at 120 and 168 hpi. FCV replicated less efficiently in ISE6 cells with recovered titres being 2–3 logs lower than the titres from BSR cells at time points 24–120 hpi (Fig. 8e). However, peak titre in this cell line was only detected at 168 hpi (7 dpi, average titre 10^4.41^/mL), suggesting that productive infection in ISE6 cells may persist past the time points sampled in this study.

### Virus-like sequences in transcriptome data of *I. holocyclus* ticks

**Fig. 9:**
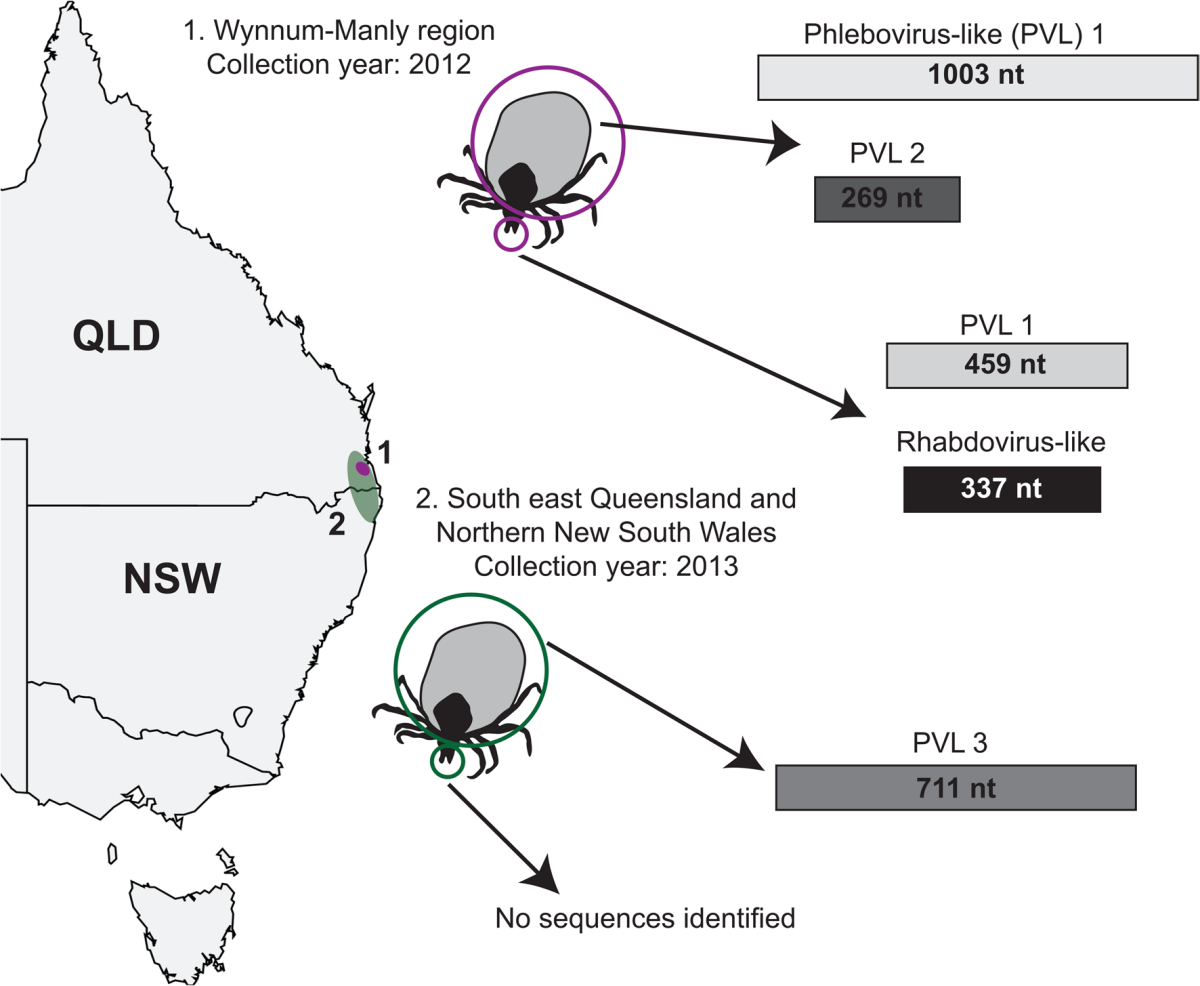
Summary of virus-like sequences identified in *I. holocyclus* transcriptome data. (1) Samples circled in magenta indicate salivary gland (heads) or viscera (bodies) data sets from ticks collected in the Wynnum-Manly region of QLD. (2) Samples circled in green indicate salivary gland or viscera data sets from pooled ticks collected throughout NSW and South-east QLD. The approximate regions of tick collections are indicated with shading in respective colours on the map. The length of each identified sequence is indicated in nucleotides (nt)

We were given access to viral sequence contigs previously assembled from transcriptome sequencing of *I. holocyclus* ticks collected in South-East Queensland (QLD) and Northern New South Wales (NSW, Table 6) [46, 54]. Our analysis focussed primarily on contigs with similarity to RNA viruses and excluded any sequences of the tick-specific IhIV which we have reported previously [34]. While IhIV sequence was detected in five of the 6 data sets [34], only three data sets yielded potential arbovirus-like sequences (Table 6, Fig. 9).

**Table 6:** Summary of *I. holocyclus* transcriptome data analysed for viral sequences

Sequencing technology	Location	Collection Year	Description of collection	Sample type
454-ion torrent	Pinjara Hills, QLD	2010	Feeding on bandicoots	Salivary glands
454-ion torrent	Suffolk Park, NSW	2010	Unfed adults	Whole ticks
Illumina^a^	Wynnum-Manly, QLD	2012	Paralysed cats and dogs	Viscera
Illumina^a^	Wynnum-Manly, QLD	2012	Paralysed cats and dogs	Salivary glands
Illumina^a^	South-east QLD and Northern NSW	2013	Paralysed cats and dogs	Viscera
Illumina	South-east QLD and Northern NSW	2013	Paralysed cats and dogs	Salivary glands

^a^Data sets containing arbovirus-like sequences described in this paper. *QLD* Queensland, *NSW* New South Wales

From the viscera of ticks collected from paralysed cats and dogs, three distinct sequences were identified which resembled the phlebovirus nucleocapsid gene (Fig. 9). These sequences will be referred to from herein as PVL (Phlebovirus-like) 1, 2 and 3. PVL1 and 2 were identified in ticks collected in the Wynnum-Manly region of QLD, while PVL3 was identified in pooled viscera of ticks collected in Northern NSW and South-east QLD (Fig. 9). Only one sequence, PVL1 was identified in datasets from paired viscera and salivary glands (Fig. 9). PVL1 and 3 showed sequence homology to two variants of Ronne virus, a novel phlebovirus identified in *I. uriae* ticks from Antarctica and Norway (Table 7) [53, 55]. These sequences also shared 66.7% nucleotide and 76.9% amino acid identity with one another. PVL2 showed homology to Timbillica virus, a novel virus identified in *I. holocyclus* ticks from Southern NSW [20] (Table 7). Finally, a small contig with similarity to the rhabdovirus RdRp gene was identified in sequence data from salivary glands, but not paired viscera, of ticks collected in Wynnum-Manly, QLD (Fig. 9, Table 7).

**Table 7:** Closest matches to *I. holocylcus* virus-like sequences by Blastx database search

Sequence name	Sequence length (nt/aa)	Blastx search result					
		Closest relative	Accession no	Gene	% ID	% QC	E-value
Rhabdo-like	337/65	Manly virus	AYP67529	RdRp	37.86	91	7e-13
PVL1	1003/237	Ronne virus	QKK82905	Nucleocapsid	38.96	69	2e-44
PVL2	269/69	Timbillica virus	AYP67565	Nucleocapsid	35.53	84	3e-05
PVL3	711/237	Ronne virus	QIS88056	Capsid	38.53	97	8e-52

### Sequence analysis of two phlebovirus-like sequences from *I. holocyclus* ticks

**Fig. 10:**
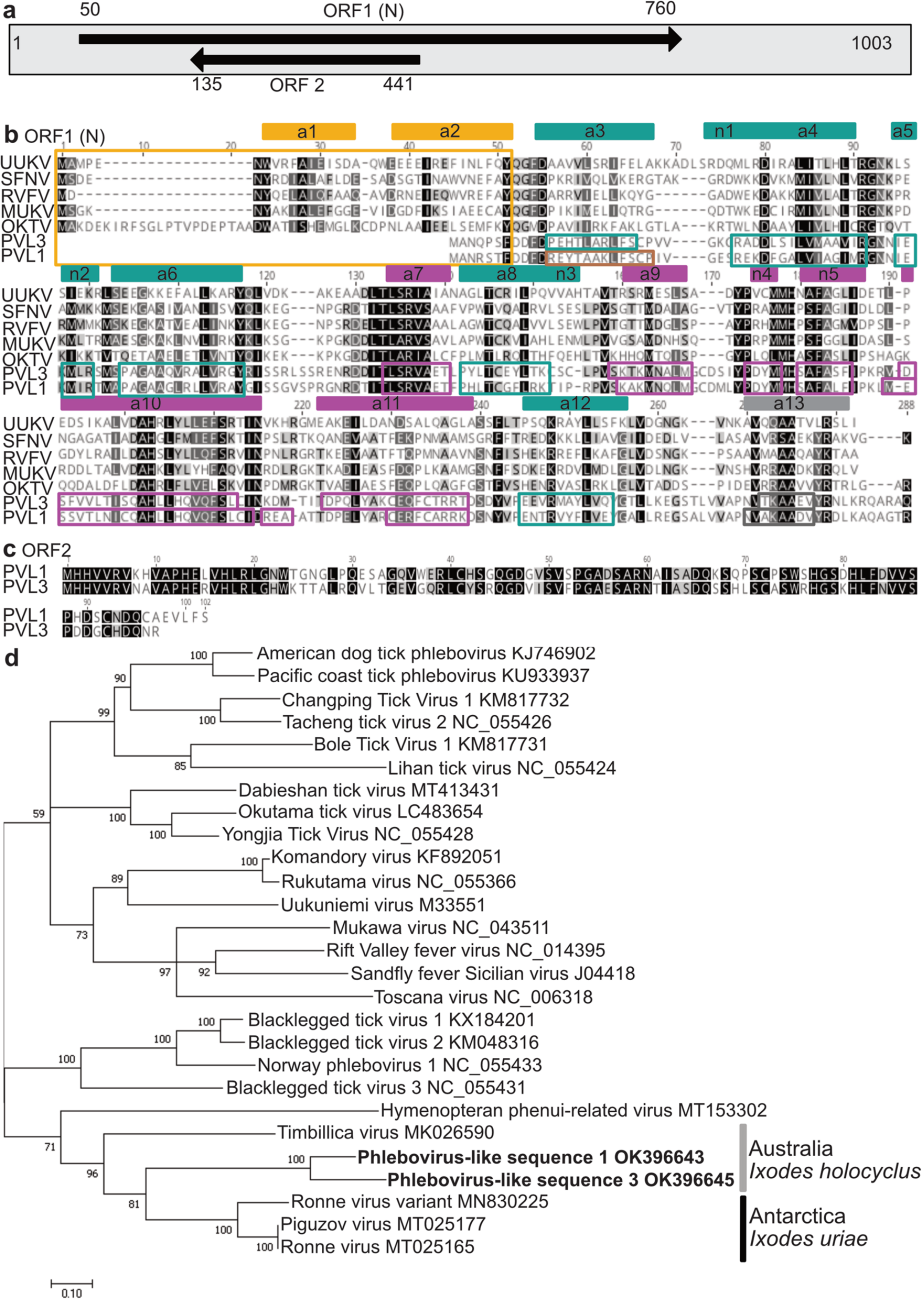
Characterisation of phlebovirus-like sequences from *I. holocyclus* ticks. **a** Schematic of the organisation of PVL1. The position and coding direction of two ORFs which were also found in PVL3 are indicated by arrows and nucleotide numbers. N, nucleocapsid-like protein gene. **b** MAFFT alignment of the 237 amino acid proteins encoded by PVL1 and PVL3 with nucleocapsid proteins of selected phleboviruses. Rectangles above sequence indicate elements of secondary structure identified for Rift Valley Fever virus (RVFV) nucleocapsid protein (alpha helices, a1–a13). Boxes enclose secondary structures predicted in PVL1 and PVL3 sequences using Phyre2. Yellow, n-terminus arm domain; green, N-lobe of globular core domain; magenta, C-lobe of globular core domain; grey, c-terminus not involved in RNA binding. Brown box at N-terminus of PVL1 indicates predicted beta strand at the expected position of alpha helix 3. UUKV, Uukuniemi virus; SFNV, Sandfly fever Naples virus; MUKV, Mukawa virus; OKTV, Okutama tick virus. **c** Alignment of a second conserved protein identified in both PVL1 and PVL3. **d** Maximum likelihood analysis of amino acid sequences of phlebovirus nucleocapsid proteins and proteins of PVL1 and PVL3. Maximum-likelihood analysis was performed using the LG + G + I model in Mega7. The percentage of trees in which the associated taxa clustered together is shown next to the branches. Grey vertical line indicates sequences identified in *I. holocyclus* ticks from Australia, black line indicates sequences from *I. uriae* ticks in Antarctica

PVL1 and 3 both contained a 711 nucleotide-long ORF encoding a 237 amino acid nucleocapsid-like protein (Fig. 10a, b). This protein shares homology at both sequence and structural levels with phlebovirus nucleocapsid (N) proteins (Fig. 10b, Additional file 1: Fig. S3). However, both proteins lacked alpha helices 1 and 2, which make up the N-terminus arm shown to be responsible for stabilisation of Rift Valley fever and Toscana virus nucleoprotein hexamers [49, 56] (Fig. 10b, Additional file 1: Fig. S3). PVL3 contained all conserved structural elements found in the globular domains of phlebovirus nucleocapsid proteins (alpha helices 3–13), while in PVL1 alpha helix 3 (a3) was substituted for a beta strand (Fig. 10b, Additional file 1: Fig. S3). PVL1 and 3 also contained a second short ORF in the reverse orientation (Fig. 10a). The resulting proteins from this ORF share 64% amino acid similarity with one another but do not show any conservation to known proteins by BLAST or HMMER database searches (Fig. 10a, c).

Maximum-likelihood analysis performed on the nucleocapsid proteins of PVL1, PVL3 and selected phleboviruses suggested that PVL1 and 3 cluster with other virus sequences identified in *I. uriae* and *I. holocyclus* ticks from the Southern hemisphere (Fig. 10d).

### Attempts to isolate viruses from *I. holocyclus* ticks

Primers were designed to test for each of the viral sequences identified above in homogenates of *I. holocyclus* ticks collected in northern NSW between 2000 and 2001. All four sequences were detected by RT-PCR in ticks of this cohort (Table 8). The amplified sequences shared high sequence similarity with the original sequences; however, attempts to amplify additional sequence from these samples using degenerate primers and next generation sequencing were unsuccessful.

**Table 8:** Detection of virus-like sequences in *I. holocyclus* homogenates

Sequence name	Accession no.	Product size (bp)	Homogenate (positive/tested)	Nucleotide similarity to original sequence (%)
Rhabdo-like	OK396646	221	4/10	100.0
PVL1	OK396643	623	3/10	99.84
PVL2	OK396644	142	5/6	100.0
PVL3	OK396645	388	5/6	99.74

As no further sequence could be amplified from homogenates, attempts were made to isolate viruses in cell culture. Three serial passages of RT-PCR positive homogenates were performed on cell lines derived from rodent (BHK), marsupial (OK) and tick (ISE6) origins. However, no viral isolates were identified by RT-PCR analysis or CPE scoring.

## Discussion

The field of tick-borne virus research in Australia is experiencing a new wave of focus due to advances in metagenomic sequencing technologies [17, 20, 57]. However, in vitro characterisation of newly identified viruses is currently lacking. A wealth of information is available from archival virus isolates, most of which were first reported between 1975 and 1985. We sought to provide additional characterisation of five tick-borne viruses isolated from seabird-associated ticks around Australia to establish a protocol for virus discovery and isolation based on the MAVRIC system [23].

We performed growth kinetics analysis on five Australian tick-borne viruses in vertebrate and tick cells. This analysis shows that all five viruses replicate rapidly and to high titres in the rodent-derived BSR cell line. The mammalian tick-borne flavivirus GGYV, orbivirus NUGV and phlebovirus CMCV replicated robustly in the *I. scapularis*-derived ISE6 cells, reaching peak titres within 1–1.5 log of their peak titre in vertebrate cells. In contrast to GGYV, the seabird tick-associated flavivirus SREV replicated to lower titres in ISE6 cells compared to BSR cells at all tested time points. Although SREV has been isolated from the Australian *Ixodes* species *I. eudyptidis*, the CSIRO 4 strain used in this study is derived from the soft tick *O. capensis* [11]. Furthermore, the closest relative of SREV, Meaban virus has been primarily isolated from *Onithodoros* species ticks possibly indicating a preference of these viruses for an *Ornithodoros* vector [58, 59]. If this is the case, an *Ornithodoros*-derived cell line may provide a better alternative for growth of these viruses [60]. Interestingly, SREV could still be recovered from ISE6 cells 4 weeks after infection suggesting an ability for this virus to persist in an *Ixodes*-derived cell line longer than GGYV, an *Ixodes* tick-borne flavivirus. FCV, a nairovirus isolated from *I. uriae*, also produced lower titres in ISE6 cells compared to BSR cells between 1 and 5 dpi. However, an increase in titre at 7 dpi may indicate that peak replication of this virus occurs after the time points sampled in this study. In line with this, growth kinetics of another *Ixodes*-borne nairovirus, Hazara virus, in ISE6 cells showed the virus reached peak titre at 8 dpi [61].

As the MAVRIC system has been successfully used for detection of mosquito-borne flaviviruses [23, 25], we first assessed its use with the tick-borne flaviviruses GGYV and SREV. Despite replication of both tick-borne flaviviruses in BSR and ISE6 cells, dsRNA could not be detected in either cell line by IFA or ELISA. This unexpected finding was confirmed using the commercial anti-dsRNA antibody J2, which has successfully been used to detect dsRNA in Langat virus-infected ISE6 and Vero cells permeabilised with 0.1% Triton X-100 [62], and TBEV-infected vertebrate cells following permeabilization with either 0.1% Triton X-100 or 0.05% saponin [63, 64]. We examined multiple methods of cell permeabilisation including 20% acetone and 0.5% Triton X-100 detergent in ELISA and 100% acetone, Triton X-114 and digitonin in IFA. All of the conditions tested in this study allowed for visualisation of dsRNA in cells infected with the mosquito-borne flavivirus WNV_KUN_, but not those infected with GGYV or SREV. Flavivirus replication requires the production of dsRNA as intermediates for the generation of nascent RNA genomes. This process is sequestered within invaginations of the host cell membranes which house the viral proteins responsible for RNA synthesis [62, 65]. Considering that this process has been demonstrated in both mosquito- and tick-borne flavivirus infection [64, 66], it seems likely that the lack of dsRNA detected in GGYV and SREV-infected cells is a result of poor exposure of the replicative intermediates during immunolabelling rather than an absence of dsRNA. Furthermore, detection of dsRNA in GGYV-infected cells at 12 hpi but not at later time points could suggest that the virus replication complexes become less permeable as infection progresses. Previously we have demonstrated that 20% acetone fixation does not sufficiently expose dsRNA during dengue virus infection; however, in this instance dsRNA could be detected following fixation with 100% acetone [23]. In lieu of MAVRIC, our antigenic analyses identified five pan-flavivirus mAbs reactive to the E-protein that can be used for detection of both a mammalian- and a seabird-associated tick flavivirus. Furthermore, we found that one mAb, M2-1E7, could be used to distinguish between the two viruses.

In contrast to our findings with SREV and GGYV, the archival tick-borne orbivirus NUGV was detectable using our anti-dsRNA mAbs from 12 hpi in vertebrate cells and 48 hpi in tick cells. In this study we also report the first genomic sequence data for NUGV. Our data shows that NUGV clusters with the GIV species, consistent with previous serological data [9]. Furthermore, NUGV shares high nucleotide (96–98%) and amino acid (97–100%) similarity with the available sequences for Sandy Bay virus (SBaV) encompassing the outer capsid protein VP5, the capping enzyme VP4 and two non-structural proteins NS2 and NS3. As these genes are typically less conserved between orbiviruses [67, 68], the high level of sequence similarity indicates that SBaV is likely a contemporary isolate of NUGV. However, sequencing of the additional genome segments of SBaV is required to confirm this.

We generated complete ORF sequences for the L and M segments of the phlebovirus CMCV and all three segments of the nairovirus FCV. Both viruses share high similarity with archival viruses isolated from Macquarie Island 27–30 years earlier [9, 10]. These data suggest that these viruses represent conventional isolates of the phlebovirus PPV and nairovirus TAGV. Furthermore, we demonstrated that serum raised to FCV detected both FCV and TAGV equally well in fixed-cell ELISA. The closeness of FCV to the original TAGV isolate is particularly interesting considering recent detections of two TAGV variants in *I. uriae* ticks from Antarctica in 2017 and 2018 which share 94–99% nucleotide identity to one another but are divergent from the prototype TAGV strain and FCV [53, 55].

Access to transcriptome sequencing of *I. holocylcus* ticks allowed us to identify four virus-like sequences. These sequences were all related to, but distinct from, sequences previously described in meta-transcriptomic surveys of *I. holocyclus* ticks collected in Sydney and Southern NSW, and *I. uriae* ticks collected in Norway and Antarctica between 2016 and 2017 [20, 53, 55]. RT-PCR analysis of *I. holocyclus* ticks collected in Northern NSW 10–12 years earlier confirmed the presence of all four sequences. However, attempts to generate additional sequence or obtain isolates in cell culture were unsuccessful. Two sequences, Phlebovirus-like (PVL) sequence 1 and 3, encoded proteins with homology to phlebovirus nucleocapsid proteins. Phylogenetic analysis of these proteins indicated that they clustered with other tick-specific phlebovirus sequences identified in the southern hemisphere. However, both proteins contained a truncated N-terminus contrasting with findings for other recently identified tick phleboviruses, which have significantly larger nucleocapsid proteins with elongated N-terminal domains [20, 53, 69]. At this stage it is unclear whether these sequences represent viruses or viral integrations. However, the presence of PVL1 in the viscera and salivary glands of ticks from Queensland is particularly interesting in the context of IhIV, which was also identified in both the salivary glands and viscera of *I. holocyclus* ticks but did not replicate in ISE6 cells [34]. Neither the four virus-like sequences described in this study nor their relatives described in [20] were identified in a recent meta-transcriptomic study performed on unfed adult *I. holocyclus* ticks collected in Northern NSW [21].

## Conclusion

In this study we performed extended characterisation on five archival Australian tick viruses in vertebrate and tick cells to discern whether our mosquito-virus discovery method could be adapted for Australian ticks. Our findings indicate that the established *I. scapularis* ISE6 cell line can support replication of Australian tick-borne arboviruses, including a flavivirus from an Argasid tick. Furthermore, we have demonstrated that a combined approach of anti-dsRNA and pan-flavivirus mAbs should allow for identification of reoviruses and flaviviruses from Australian ticks. However, the ability of a northern hemisphere-derived tick cell line to support replication of Australian tick-specific viruses is still unclear. Cell lines derived from Australian native ticks may be necessary for future characterisation of tick-specific viruses in this region. Finally, next generation sequencing of NUGV, CMCV and FCV further highlights the need for sequencing of archival viruses to prevent isolates of a single virus receiving multiple names.

## Supplementary Information

**Additional file 1: Table S1.** List of reagents used in complete ISE6 cell media available in Australia. Product codes and company from which each reagent was purchased are described. **Figure S1.** Double-stranded RNA immunolabelling in flavivirus-infected cells after permeabilization with digitonin. Immunofluorescence images of BSR cells infected with tick-borne flaviviruses Saumarez Reef virus (SREV), Gadgets Gully virus (GGYV) or mosquito-borne West Nile virus subtype Kunjin (WNV_KUN_). Cells were fixed with 4% formaldehyde and permeabilised using a solution of 0.5% *w/v* digitonin prior to labelling with anti-dsRNA monoclonal antibodies (**a**) MAVRIC or (**b**) J2. Images were taken on a Zeiss LSM 510 microscope at 20 × magnification. Blue, nuclei; green, dsRNA. **Figure S2.** Phylogenetic relationship of NUGV and members of the genus Orbivirus. Phylogenetic analysis of the nucleotide coding sequences of (**a**) T2 and (**b**) VP7 (T13) genes. Phylogenetic analyses were performed in MEGA7 using the JC + I model. Bold branches denote members of the tick-borne orbivirus group. The percentage of trees in which the associated taxa clustered together is shown next to the branches. **Figure S3.** Tertiary structure predictions of Phlebovirus-like nucleocapsid sequences. Predicted protein structure of nucleocapsid-like proteins encoded by (**a**) PVL1 and (**b**) PVL3. Protein structures were predicted using Phyre2 based on alignment with Toscana virus N protein (100% confidence). Predicted domains are colour coded as follows: green, N-lobe of globular core domain; magenta, C-lobe of globular core domain; grey, c-terminus not involved in RNA binding; Brown, N-terminus of PVL1 contains a predicted beta strand in lieu of alpha helix 3; black, N-terminus arm domain.

**Additional file 2: Dataset S1.** Mass spectrometry analysis of Finch Creek virus proteins. Excel file containing the results of analysis of mass spectrometry data from two proteins of Finch Creek virus identified in SDS-PAGE. This file contains the peptide sequences identified in each sample.

## Data Availability

The sequences described in this paper have been submitted to Genbank under the following accession numbers: NUGV (OK396633–OK396642), CMCV (OK493375 and OK493376), FCV (OK493377–OK493379), *I. holocyclus* virus-like sequences (OK396643–OK396646). The *I. holocyclus* transcriptome data sets have been published previously and can be found at: https://www.ncbi.nlm.nih.gov/genbank/, GIBQ01000000.1.

## Abbreviations

DSCATT: Debilitating symptoms complex attributed to ticks
CSIRO: Commonwealth Science and Industrial Research Organisation
SREV: Saumarez Reef virus
GGYV: Gagdets Gully virus
WNV_KUN_: West Nile virus subtype Kunjin
CMCV: Catch-me-cave virus
PPV: Precarious Point virus
FCV: Finch Creek virus
TAGV: Taggert virus
NUGV: Nugget virus
SBaV: Sandy Bay virus
GIV: Great Island virus
MAVRIC: Monoclonal antibodies to viral RNA intermediates in cells
SDS-PAGE: Sodium dodecyl sulphate-polyacrylamide gel electrophoresis
PBS: Phosphate-buffered saline
PBS-T: Phosphate-buffered saline with 0.05% *v/v* Tween-20
VINHV: Vinegar Hill virus
ISE6: *Ixodes scapularis* Cell line
OK: Opossum kidney cell line
BHK: Baby hamster kidney cell line
BSR: Clone of BHK cell line
Vero: African green monkey kidney cell line
C6/36: *Aedes albopictus* Mosquito cell line
PVL: Phlebovirus-like sequence
QLD: Queensland
NSW: New South Wales
