# Supplementary material for: Extended characterisation of five archival tick-borne viruses provides insights for virus discovery in Australian ticks
Source: Parasit Vectors. 2022 Feb 18;15:59. doi: 10.1186/s13071-022-05176-z (PMC8857802; doi:10.1186/s13071-022-05176-z)
Supplement: Supplementary file 1 — Additional file 1: Table S1. List of reagents used in complete ISE6 cell media available in Australia. Product codes and company from which each reagent was purchased are described. Figure S1. Double-stranded RNA immunolabelling in flavivirus-infected cells after permeabilization with digitonin. Immunofluorescence images of BSR cells infected with tick-borne flaviviruses Saumarez Reef virus (SREV), Gadgets Gully virus (GGYV) or mosquito-borne West Nile virus subtype Kunjin (WNVKUN). Cells were fixed with 4% formaldehyde and permeabilised using a solution of 0.5% w/v digitonin prior to labelling with anti-dsRNA monoclonal antibodies (a) MAVRIC or (b) J2. Images were taken on a Zeiss LSM 510 microscope at 20 × magnification. Blue, nuclei; green, dsRNA. Figure S2. Phylogenetic relationship of NUGV and members of the genus Orbivirus. Phylogenetic analysis of the nucleotide coding sequences of (a) T2 and (b) VP7 (T13) genes. Phylogenetic analyses were performed in MEGA7 using the JC + I model. Bold branches denote members of the tick-borne orbivirus group. The percentage of trees in which the associated taxa clustered together is shown next to the branches. Figure S3. Tertiary structure predictions of Phlebovirus-like nucleocapsid sequences. Predicted protein structure of nucleocapsid-like proteins encoded by (a) PVL1 and (b) PVL3. Protein structures were predicted using Phyre2 based on alignment with Toscana virus N protein (100% confidence). Predicted domains are colour coded as follows: green, N-lobe of globular core domain; magenta, C-lobe of globular core domain; grey, c-terminus not involved in RNA binding; Brown, N-terminus of PVL1 contains a predicted beta strand in lieu of alpha helix 3; black, N-terminus arm domain. [file 13071_2022_5176_MOESM1_ESM.pdf]

Table S1

Component	Supplier	Product code	Details
LipiMAX bovine lipoprotein solution	Selborne Biological	ABL-03	Low endotoxin
Tryptose phosphate broth	Oxoid, Thermo Fisher	CM0283B	Bacteria and cell culture
L-aspartic acid	Sigma-Aldrich	A9256	≥98% pure
L-glutamine 200mM solution	Sigma-Aldrich	G7513	sterile filtered, cell culture
L-Glutamine	Sigma-Aldrich	G3126	99% pure
L-proline	Alfa Aesar, Thermo Fisher	A10199	99% pure
L-glutamic acid	Sigma-Aldrich	G1626	≥99% pure (HPLC grade)
A-ketoglutaric acid	Sigma-Aldrich	K1750	98.5% pure
D-glucose	ChemSupplyAustralia	GL01251000	extra pure, anhydrous
NaOH	ChemSupplyAustralia	SA000	For pH
**Stock solution A**			
Cobalt (II) chloride hexahydrate	Supleco, Merck Millipore	1.02539.0100	6H2O
Copper (II)sulfate	Sigma-Aldrich	C1297	Anhydrous, >99% pure
Manganese (II) sulfate monohydrate	Sigma-Aldrich	M7634	>99% pure
Zinc sulfate heptahydrate	Sigma-Aldrich	221376	ACS reagent, 99%
**Stock solution B**			
Sodium molybdate dihydrate	Sigma-Aldrich	M1003	≥99.5% pure
**Stock solution C**			
Sodium Selenite	Sigma-Aldrich	S5261	Anhydrous, cell culture grade
**Stock solution D**			
L-glutathione, reduced	Sigma-Aldrich	G4251	98% pure
L-Ascorbic acid	Vetec, Sigma-Aldrich	V800232	>99.5% pure
Iron (II) sulfate	Sigma-Aldrich	F7002	heptahydrate
**Vitamin Stock**			
p-aminobenzoic acid	Sigma-Aldrich	A9878	(4-aminobenzoic acid) >99%
Vitamin B12 (Cyanocobalamin)	Sigma-Aldrich	V6629	Cell culture grade, >98%
D-Biotin	Sigma-Aldrich	47868	Analytical reagent

Figure S1

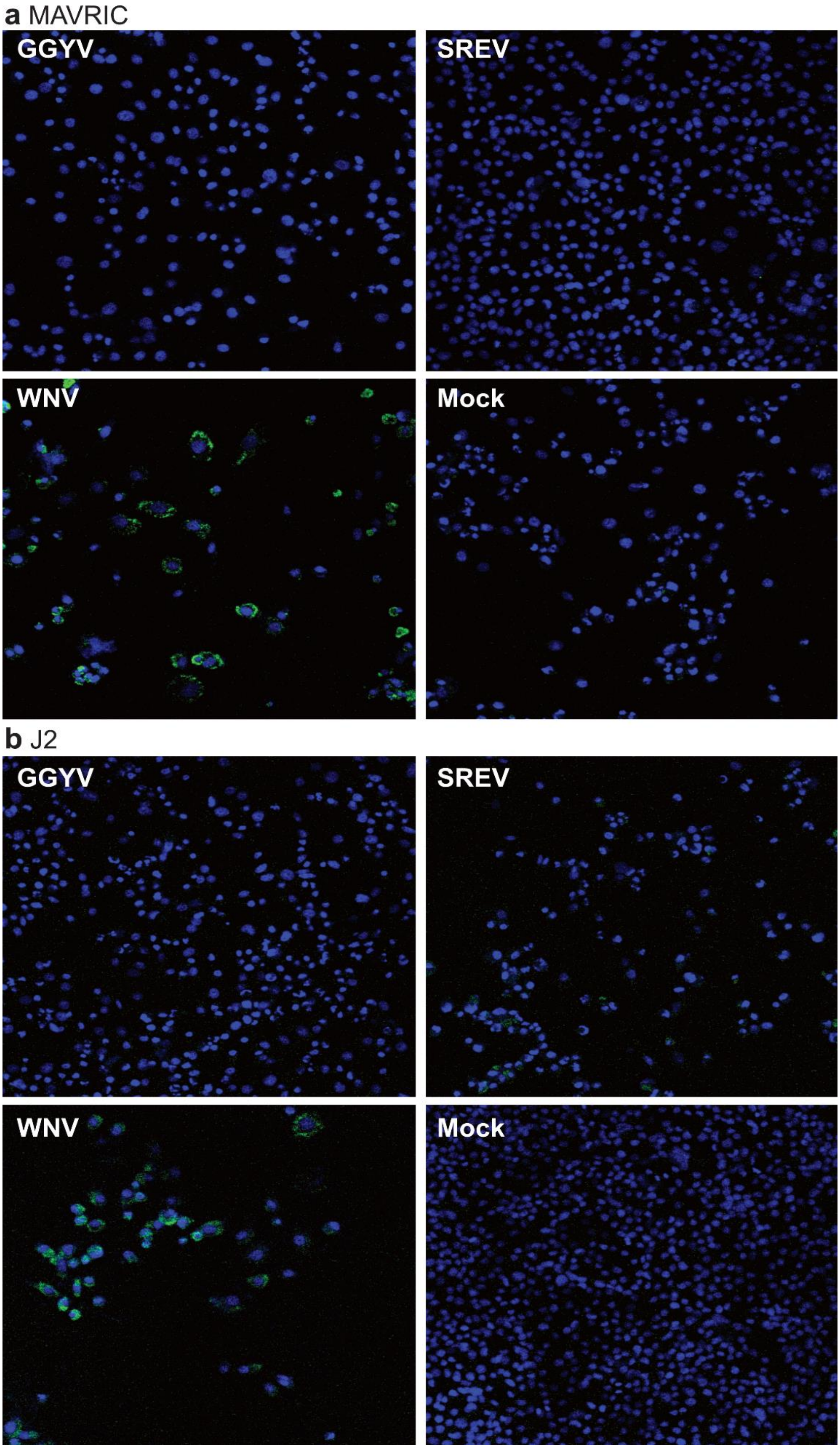

Figure S2

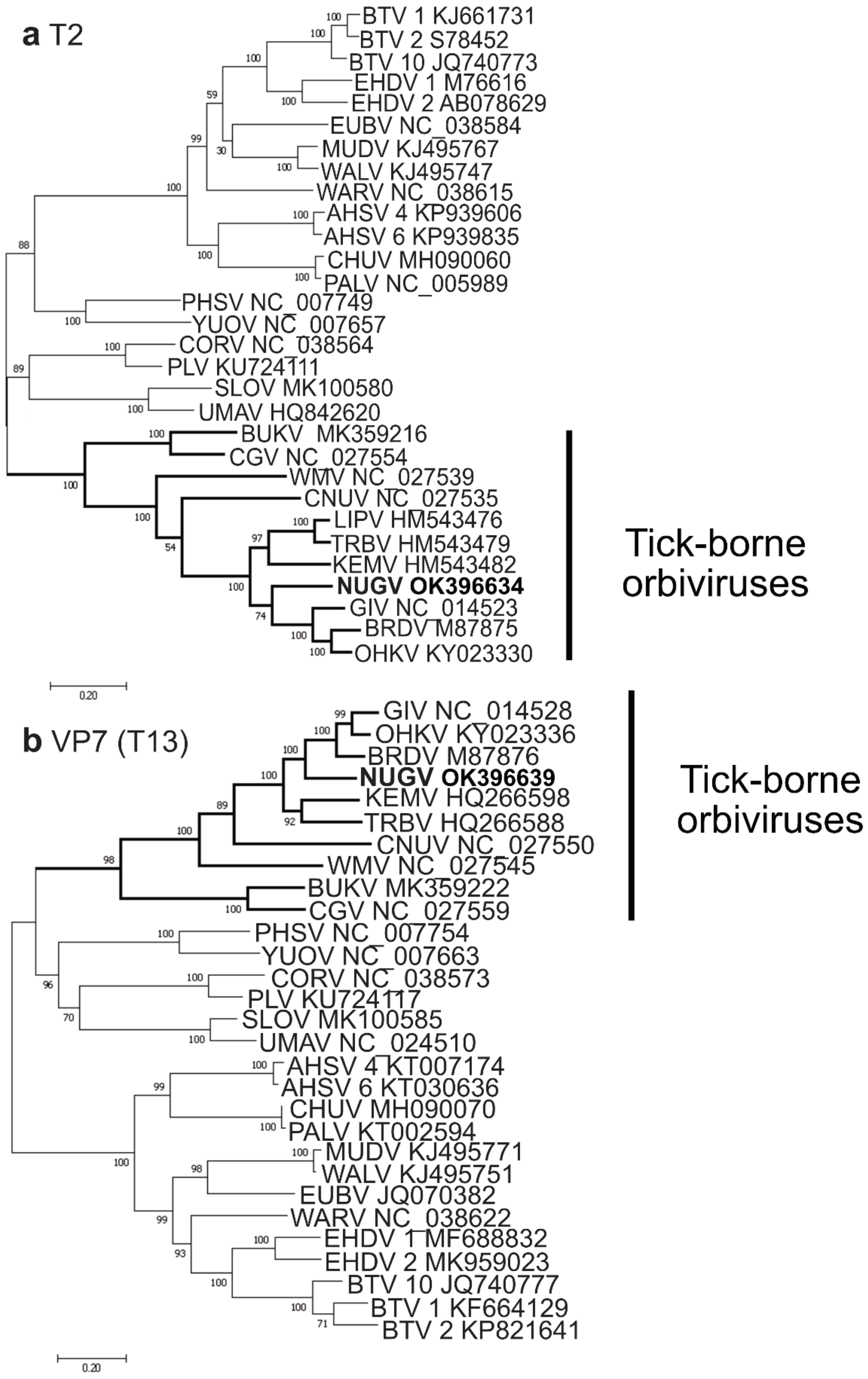
a T2
BTV 1 KJ661731
BTV 2 S78452
BTV 10 JQ740773
EHDV 1 M76616
EHDV 2 AB078629
EUBV NC_038584
MUDV KJ495767
WALV KJ495747
WARV NC_038615
AHSV 4 KP939606
AHSV 6 KP939835
CHUV MH090060
PALV NC_005989
PHSV NC_007749
YUOV NC_007657
CORV NC_038564
PLV KU724111
SLOV MK100580
UMAV HQ842620
BUKV MK359216
CGV NC_027554
WMV NC_027539
CNUV NC_027535
LIPV HM543476
TRBV HM543479
KEMV HM543482
NUGV OK396634
GIV NC_014523
BRDV M87875
OHKV KY023330
Tick-borne orbiviruses
0.20
b VP7 (T13)
GIV NC_014528
OHKV KY023336
BRDV M87876
NUGV OK396639
KEMV HQ266598
TRBV HQ266588
CNUV NC_027550
WMV NC_027545
BUKV MK359222
CGV NC_027559
PHSV NC_007754
YUOV NC_007663
CORV NC_038573
PLV KU724117
SLOV MK100585
UMAV NC_024510
AHSV 4 KT007174
AHSV 6 KT030636
CHUV MH090070
PALV KT002594
MUDV KJ495771
WALV KJ495751
EUBV JQ070382
WARV NC_038622
EHDV 1 MF688832
EHDV 2 MK959023
BTV 10 JQ740777
BTV 1 KF664129
BTV 2 KP821641
Tick-borne orbiviruses
0.20

Figure S3

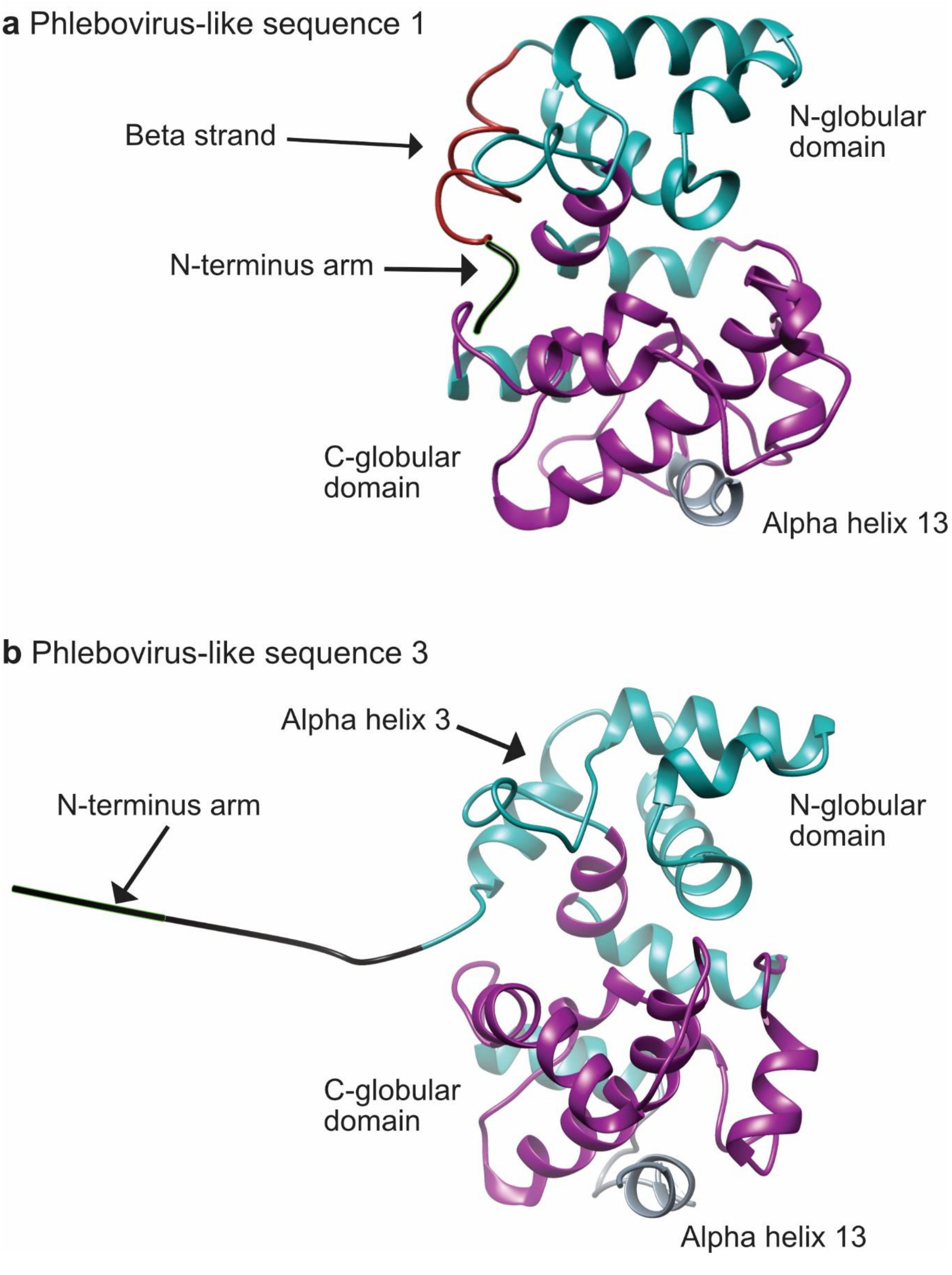
a Phlebovirus-like sequence 1
Beta strand
N-globular
domain
N-terminus arm
C-globular
domain
Alpha helix 13
b Phlebovirus-like sequence 3
Alpha helix 3
N-terminus arm
N-globular
domain
C-globular
domain
Alpha helix 13
